# A Computational Model of the Cholinergic Modulation of CA1 Pyramidal Cell Activity

**DOI:** 10.3389/fncom.2020.00075

**Published:** 2020-09-04

**Authors:** Adam Mergenthal, Jean-Marie C. Bouteiller, Gene J. Yu, Theodore W. Berger

**Affiliations:** Biomedical Engineering Department, Center for Neural Engineering, University of Southern California, Los Angeles, CA, United States

**Keywords:** hippocampus, acetylcholine, CA1, muscarinic, compartmental model, pyramidal, computational

## Abstract

Dysfunction in cholinergic modulation has been linked to a variety of cognitive disorders including Alzheimer's disease. The important role of this neurotransmitter has been explored in a variety of experiments, yet many questions remain unanswered about the contribution of cholinergic modulation to healthy hippocampal function. To address this question, we have developed a model of CA1 pyramidal neuron that takes into consideration muscarinic receptor activation in response to changes in extracellular concentration of acetylcholine and its effects on cellular excitability and downstream intracellular calcium dynamics. This model incorporates a variety of molecular agents to accurately simulate several processes heretofore ignored in computational modeling of CA1 pyramidal neurons. These processes include the inhibition of ionic channels by phospholipid depletion along with the release of calcium from intracellular stores (i.e., the endoplasmic reticulum). This paper describes the model and the methods used to calibrate its behavior to match experimental results. The result of this work is a compartmental model with calibrated mechanisms for simulating the intracellular calcium dynamics of CA1 pyramidal cells with a focus on those related to release from calcium stores in the endoplasmic reticulum. From this model we also make various predictions for how the inhibitory and excitatory responses to cholinergic modulation vary with agonist concentration. This model expands the capabilities of CA1 pyramidal cell models through the explicit modeling of molecular interactions involved in healthy cognitive function and disease. Through this expanded model we come closer to simulating these diseases and gaining the knowledge required to develop novel treatments.

## 1. Introduction

**Figure 1:**
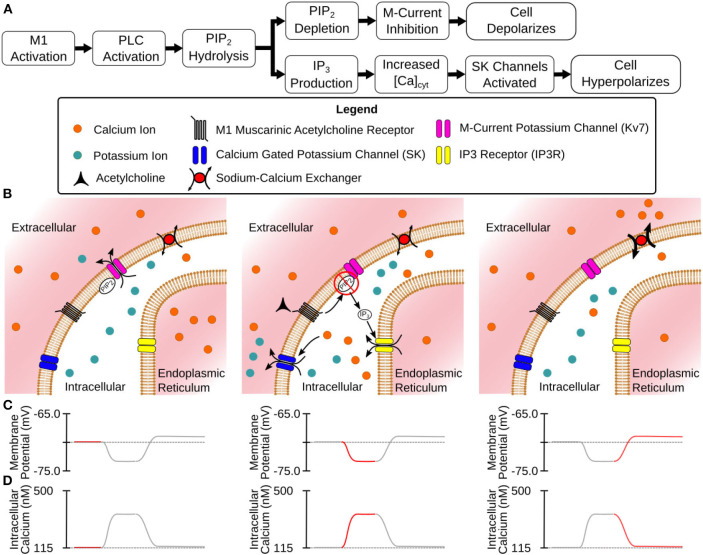
Mechanisms of CA1 pyramidal cell modulation by M1 MAChRs. **(A)** Flowchart description of the steps between M1 activation and modulated membrane potential. **(B)** Illustrated stages of activation: (Left) Before activation the cell is at rest with Kv7 channels open. (Center) M1 activation leads to hydrolysis of PIP_2_ from cell membrane (inhibiting Kv7 channels) and the release of intracellular Ca^2+^ (activating SK channels) through the generation of IP_3_. (Right) As Ca^2+^ is extruded from the intracellular space SK channels close while Kv7 channels remain closed. **(C)** Membrane potential at different stages of activation. **(D)** Intracellular calcium levels at different stages of activation.

Acetylcholine (ACh) directly modulates the activity of neurons within every subregion of the hippocampus, including both principal neurons and interneurons (Aznavour et al., 2002; Takács et al., 2018). The dense distribution of the cholinergic terminals within the hippocampus suggests that this neurotransmitter plays an important role in healthy hippocampal functioning. This important role is further evidenced by the correlation of dysfunctions reported in cholinergic terminals with cognitive impairment. The progression of Alzheimer's disease (AD) has long been associated with the decline of cholinergic markers in the hippocampus (Schliebs and Arendt, 2011). Other cognitive disorders such as depression and schizophrenia are also associated with alterations in cholinergic disregulation (Higley and Picciotto, 2014). On an even broader scale, changes in cholinergic expression are associated with the cognitive decline due to advanced age (Schliebs and Arendt, 2011). The variety of cognitive dysfunctions related to ACh suggests that it plays not only an important, but a complex role. In a 2-year double-blind study, 35% of patients taking an acetylcholinesterase inhibitor to slow cognitive decline due to AD had a recurrence of major depressive episodes vs. 19% of those on a placebo (Reynolds et al., 2011). In other words, a drug meant to counteract one form of cholinergic dysfunction exacerbated a separate form of cholinergic dysfunction. Developing better treatments for these disorders requires a better understanding of the dynamics of healthy cholinergic modulation. Currently, ACh is understood to play a role in a variety of cognitive processes. We will summarize some of these effects briefly but for fuller reviews (see Dannenberg et al., 2017; Solari and Hangya, 2018). Acetylcholine has long been understood to be involved in the generation of theta oscillations (4–12 Hz) in the hippocampus. Theta oscillations are theorized to organize memory encoding and retrieval into distinct phases (Hasselmo et al., 2002). Acetylcholine seems to be involved with the generation of the lower frequency portion of theta oscillations, as these frequencies can be blocked by the cholinergic receptor antagonist atropine (Kramis et al., 1975). On a behavioral level, the blockade of cholinergic receptors in animal models leads to a variety of memory deficits involving both spatial navigation and the acquisition of conditioned fear responses (Jiang et al., 2016; Solari and Hangya, 2018). These effects result from the activation of a variety of cholinergic receptors in the hippocampus. These receptors can be sorted into two types. The first type, nicotinic receptors, act as ionotropic receptors and allow the passage of ions through the plasma membrane. In the CA1, nicotinic receptors primarily modulate interneuron activity (McQuiston, 2014), but they also appear in low densities on pyramidal cells (Kalappa et al., 2010). The second type, muscarinic receptors, have a much larger effect in modulating CA1 pyramidal cell activity (Dasari and Gulledge, 2011). These receptors are G protein coupled receptors with their activation setting off a cascade of intracellular reactions. Among the five subtypes of muscarinic acetylcholine receptors (mAChRs), the subtypes that primarily modulate CA1 pyramidal activity are the M1 and M4 mAChRs. M4 mAChRs suppress glutamatergic release from excitatory synapses originating from the CA3 subregion (Dasari and Gulledge, 2011). M1 mAChRs are present throughout the cell's morphology and alter its overall excitability along with altering the intracellular calcium dynamics (Dasari and Gulledge, 2011). Thus, the M1 mAChRs are responsible for the majority of the cholinergic response in this cell type. The M1 mAChR, as a G-protein coupled receptor, activates a cascade of intracellular reactions (Falkenburger et al., 2010a,b). It is through these reactions that the M1 receptor is able to modulate the behavior of a variety of ion channels. Teithehe M-current was given that name due to muscarinic receptors suppressing its activity (Brown and Adams, 1980). Inhibition of this current in CA1 pyramidal cells through bath application of the M-current antagonist XE991 lead to a depolarized resting membrane potential and increased spiking activity (Shah et al., 2008). This current was also shown to be inhibited after bath application of the muscarinic agonist Oxotremorine-M (Oxo-M) (Carver and Shapiro, 2019). The channels responsible for the M-current, Kv7 Potassium channels, require phosphatidylinositol 4,5-bisphosphate (PIP_2_) in the cell membrane to maintain its open state. M1 activation leads to the activation of phospholipase C (PLC) which hydrolyzes PIP_2_ into inositol(1,4,5)triphosphate (IP_3_) and diacylglycerol (Falkenburger et al., 2010a,b). It is through this depletion of PIP_2_ that mAChRs inhibit the M-current. Also, by producing IP_3_, M1 receptors trigger the release of calcium from the endoplasmic reticulum (ER) via IP_3_ receptors. This leads to an increase in intracellular calcium which activates calcium dependent potassium (SK) channels. In CA1 pyramidal cells M1 activation is followed by a hyperpolarization which is able to inhibit action potentials. These hyperpolarizations can be blocked through the application of apamin, an SK channel antagonist (Dasari and Gulledge, 2011). Figure 1 provides both a flowchart and a cartoon illustrate these processes. One long term goal of our lab has been to create a large scale model of the hippocampus and through this model, gain a better understanding of the underlying dynamics of this system (Hendrickson et al., 2015), thereby facilitating the development of better treatments (electrical or pharmaceutical) to alleviate hippocampal dysfunctions. Experimental evidence has demonstrated that cholinergic modulation plays an important role in controlling the dynamics of this system. This has driven the development of this single cell model, which will act as a foundation for integrating cholinergic modulation into our efforts for a large-scale hippocampal model. We have chosen to build the single cell model on a biophysically realistic basis wherever possible. This is for two reasons. First, the collection of experimental data for calibrating a model gives a perspective on the depth of understanding and raises questions to guide further *in vitro* or *in vivo* experimental efforts. Second, the inclusion of biochemical mechanisms allows for broad parametric manipulations which (i) facilitate the simulation of pathological processes and disease states and (ii) provide useful insights for the identification and development of novel treatment options. By creating a biophysically realistic model, we have developed a tool that allows more cohesive collaboration with other experimental efforts. What follows is a description of a model for the cholinergic modulation of the somatic activity of pyramidal cells within the CA1 region of the hippocampus. Within the hippocampus, this cell type is the most studied in terms of cholinergic modulation and will constitute a solid foundation for the construction of larger cell network models.

## 2. Materials and Methods

**Figure 2:**
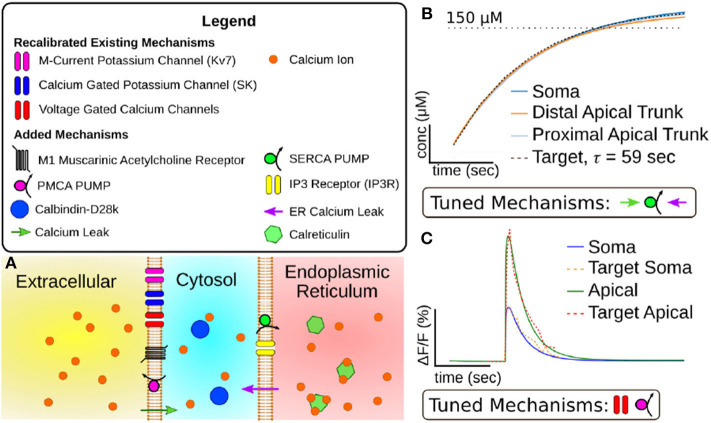
**(A)** Visualization of mechanisms included in expanded CA1 pyramidal cell calcium model. **(B)** Results of tuning model mechanisms that replenish calcium stores in the endoplasmic reticulum without action potentials. Plotted value is simulated calcium stored in endoplasmic reticulum over time (x-axis = 50 s, y-axis = 50 μM). Target data is based on experiments in Garaschuk et al. (1997). **(C)** Results of tuning model mechanisms that determine calcium dynamics following an action potential. Plotted value is the percent change in fluorescence of a simulated calcium indicator (OGB-1) over time (Scale: x-axis = 1 s, y-axis= 10% change in fluorescence). Target data is based on results from Power and Sah (2002).

The primary task of this research was to evaluate and bring together a variety of mechanisms and models to accurately capture the dynamic response of CA1 pyramidal cells to acetylcholine. As a starting point, we used a compartmental model of the CA1 pyramidal cell (mpg141209_A_idA as downloaded from ModelDB) (Migliore et al., 2018) previously developed for the NEURON simulation environment (Carnevale and Hines, 2006). We chose to use this simulation environment as its RXD module (McDougal et al., 2013) allowed us to efficiently expand the model's intracellular calcium mechanisms. The code for these simulations was developed in the Python programming language. The base model included mechanisms for the M-current, SK channels, voltage-gated calcium channels (VGCC). Entry through VGCCs was the only mechanism through which intracellular calcium increased, while calcium efflux was simulated as an exponential decay of the intracellular calcium to its resting value. As one of the focuses of this work was to simulate intracellular calcium release we needed to insert and calibrate all of the mechanisms for simulating the storage and release of calcium from the endoplasmic reticulum, buffering the intracellular calcium concentration, and extrusion of excess calcium into the extracellular space. Without these mechanisms, none of the inhibitory effects seen in Figure 1 could be replicated. These calcium mechanisms were only expanded in the sections that comprise the soma and the first 200 μm of the apical dendritic trunk. Figure S1 illustrates which sections within the full morphology were given expanded calcium mechanisms. One reason for the decision to only expand the calcium model into these sections was that the calcium dynamics in these regions are the most studied due their diameters being large enough for calcium imaging using fluorescent dyes. Second, cholinergic modulation in synaptic spines seems to play a role in plasticity (Dennis et al., 2016). However, plasticity in these synapses is also dependent upon postsynaptic spiking activity. To properly simulate how plasticity is altered by cholinergic modulation requires we first make a working model of how cholinergic modulation alters cell excitability and spike generation. Finally, the mechanisms of action differ between synaptic and somatic modulation. For instance, the hyperpolarization seen at the soma is due to the activation of SK channels as evidenced by its blockade by apamin (Dasari and Gulledge, 2011), while synaptic cholinergic modulation has been tied to the inhibition of SK channels (Buchanan et al., 2010). Calibrating these differing mechanisms requires a separate series of simulations and would be best explained in a separate work. Our goal in selecting additional mechanisms was to create a relatively simple model capable of replicating intracellular calcium dynamics. Disease and age have been reported to alter several of the mechanisms included (e.g., calcium buffering; Gant et al., 2006; Oh et al., 2013). By incorporating mechanistic models for these altered states, we can explore how the cell behavior changes, and how these changes impact network-level outcomes. Of importance, fidelity to the biochemistry of the intracellular space must be balanced against the realities of computational modeling. A model that includes all of the known molecular interactions would have too many parameters to constrain with the available experimental evidence. Additionally, simulations using this model would be computationally expensive even for a single cell model. In addition, our goal of including this model into large scale network simulations only exacerbates this limitation. We have thus strived to include the minimum collection of mechanisms that is necessary for capturing cholinergic modulation in the soma and apical trunk. Expanding the model to other regions and to include other mechanisms will be performed in subsequent work. A visualization of the mechanisms in the expanded calcium model can be found in Figure 2, while the concentrations and kinetic parameters for these mechanisms can be found in Tables S1, S2, respectively. The addition of a mechanism often required constraining parameter values to properly replicate experimental results. In order to simplify the calibration process, the mechanisms were divided into groups based upon region of action (e.g., endoplasmic reticulum vs. intracellular). These groups were then calibrated in a specific order, starting from protocols that required the smallest number of mechanisms and comprised a minimum number of interdependent parameters. For example, the rate at which the endoplasmic reticulum (ER) regains depleted calcium at rest is based upon the balance between the rate of calcium uptake from sarco/endoplasmic reticulum calcium pumps (SERCA) vs. the rate of calcium leakage from the ER. Since the conductance of VGCCs does not factor into this result it can be ignored. Conversely, replicating intracellular calcium transients after an action potential requires constraining parameters for VGCC conductance and calcium extrusion, in addition to SERCA and ER leak flux, as ER calcium sequestration alters the dynamics in the intracellular space. Since we could relatively isolate the ER mechanisms, those parameters were calibrated first. This simplified the calibration of later parameters based on results that depend on more mechanisms. The following sections describe the mechanisms that were implemented and the experimental data from which constrained these parameter values.

### 2.1. Calibrating the Endoplasmic Reticulum

The first step in creating the model was to calibrate the parameters pertaining to the ER. We chose to model the ER as an idealized 10% of the intracellular volume to avoid explicitly modeling the intricate and dynamic geometry of the ER. Reconstructions of the ER in CA1 pyramidal cells have focused on the organelle's volume in either the dendritic branches or synaptic spines while ignoring the volume of the ER in the soma and apical trunk. Using smaller values for the percentage of ER volume, such as those found in dendritic reconstructions (2–8% of dendrite volume) (Spacek and Harris, 1997), decreased the capacity of calcium storage such that the model could not replicate the amplitude of calcium release events. The 10% value is therefore a compromise that allows larger intracellular calcium release events while remaining near the experimentally measured range. The resting concentration inside the ER was initialized at 175 μM (Solovyova et al., 2002). For the initial calibration, there were three mechanisms that defined the ER calcium dynamics: calreticulin (CALR) concentration, SERCA pumps, and calcium leak. The inositol(1,4,5)triphosphate receptor mechanism (IP_3_R) was calibrated at a later stage as the IP_3_R model produced negligible currents at resting IP_3_ concentrations. CALR acts as the major calcium buffer in the lumen of the ER and its concentration defines the amount of buffered calcium reserves for a given lumenal calcium concentration. We used the CALR kinetics and concentration found in an earlier ER model (Doi et al., 2005).

Expressions (1) and (2) were used for the SERCA pump mechanism while Expression (3) was the formula used to calculate the leak of calcium from the ER into the cytosol. Expression (4) shows the chemical formula used for CALR binding to calcium.

(1) $$\begin{array}{l} Ca_{cyt}^{2+}\overset{k_{f}^{S}}{\to }Ca_{ER}^{2+} \end{array}$$

(2) $$\begin{array}{l} k_{f}^{S}=\frac{g_{S}\cdot {\left[Ca_{cyt}^{2+}\right]}^{2}}{{\left[Ca_{cyt}^{2+}\right]}^{2}+0.0013^{2}} \end{array}$$

(3) $$\begin{array}{l} Ca_{ER}^{2+}\overset{k_{f}^{leak\;ER}}{\to }Ca_{cyt}^{2+} \end{array}$$

(4) $$\begin{array}{l} \text{CALR}+{\text{Ca}}^{2}+\overset{k_{f}^{calr}}{\underset{k_{b}^{calr}}{⇌}}\text{CALRCa} \end{array}$$

From these mechanisms we calibrated two parameters, *g*_*S*_, the SERCA conductance, and $k_{f}^{leak\;ER}$, the rate of leakage from the ER. Due to the model ER not having a set geometry it also lacks a set surface area. Therefore these mechanisms were implemented as direct fluxes between the two volumes without consideration of surface density. To constrain the SERCA and leak mechanisms, we used an experimental result (Garaschuk et al., 1997) for which the return of $Ca_{ER}^{2+}$ to resting concentrations was fit with an exponential function with a time constant of 59 s. This time constant along with the experimental $Ca_{ER}^{2+}$ resting concentration gave us a target with which we manually calibrated *g*_*S*_ and $k_{f}^{leak\;ER}$. The results of this calibration are illustrated in Figure 2B.

### 2.2. Calibrating Intracellular Calcium and Indicator Model

With the ER related mechanisms calibrated, we moved to calibrating the mechanisms related to the intracellular space. The major mechanisms of interest in this portion of the model pertain to the extrusion of excess calcium into the extracellular space [i.e., plasma membrane calcium pumps (PMCA)] and the conductance of VGCCs. However, experimental evidence to constrain these parameters required the addition of mechanisms to replicate experiments using fluorescent calcium indicators. Fluorescence measurements constitute the primary method to visualize calcium dynamics. However these indicators act as a high affinity calcium buffer and alter the very dynamics they are supposed to report. We therefore included mechanisms to simulate the binding of calcium to Oregon Green BAPTA-1 (OGB-1), as this was the indicator used in the experimental results we sought to replicate. The kinetic parameters we used for the OGB-1 mechanism were based on measurements in an intracellular environment as interactions with intracellular ions can change the affinity from its reported *in vitro* value (Thomas et al., 2000). Expression (5) was the chemical formula used for the binding of calcium to OGB-1. According to the product information sheet OGB-1 bound to calcium fluoresces 14 times the rate of the unbound state (Molecular Probes, 2005). We used this fact to create Expression (6), which provides a method to calculate the simulated fluorescence. With this mechanism we could use fluorescence experiments that used this calcium indicator to constrain the other intracellular mechanisms. This OGB-1 mechanism for creating a simulated fluorescence was only used in this portion of the calibration process and was not included in later parameter calibrations.

(5) $$\begin{array}{l} \text{OGB}+{\text{Ca}}^{2}+\overset{k_{f}^{ogb1}}{\underset{k_{b}^{ogb1}}{⇌}}\text{OGBCa} \end{array}$$

(6) $$\begin{array}{l} F=f_{mult}\cdot OGB1Ca+OGB1 \end{array}$$

Deciding what concentration of Calbindin-D28k (CB) to use in our simulations was another obstacle. The presence of CB is among the ways that CA1 pyramidal cells display heterogeneity, with only around 50% of cells expressing this protein (Müller et al., 2005). Expression of CB is not correlated with the bursting/regular firing characteristic that serves as the major dichotomy within CA1 pyramidal cells (Baimbridge et al., 1991), so the spiking response of base compartmental model could not be used as a constraint. Additionally CB is mostly mobile, so a large portion of CB likely diffused out of the cell and into the electrodes used to inject the fluorescent indicators as demonstrated in Müller et al. (2005). These factors make it difficult to have full confidence in the intracellular concentration of CB during the fluorescent measurements we used to calibrate the parameters. In simulations replicating fluorescent data, we assumed these cells did express this protein but that the concentration was diminished. We set the diminished concentration to 20% of its regular value as that proportion was estimated to be immobile in neurons (Schmidt et al., 2005). Expression (7) is the chemical formula for the binding of CB to calcium.

(7) $$\begin{array}{l} \text{CB}+{\text{Ca}}^{2}+\overset{k_{f}^{cb}}{\underset{k_{b}^{cb}}{⇌}}\text{CBCa} \end{array}$$

Expression (8) describes the binding of pmca to calcium while Expression (9) describes the release of calcium into the extracellular volume. This series of reactions describes how PMCA acts as the mechanism for the extrusion of calcium from the cytosol to the extracellular space.

(8) $$\begin{array}{ll} \text{PMCA}+{\text{Ca}}^{2}+ & \overset{k_{f}^{pmca\;ca}}{\underset{k_{b}^{pmca\;ca}}{⇌}}\text{PMCACa} \end{array}$$

(9) $$\begin{array}{ll} \text{PMCACa} & \overset{k^{pmca\;rel}}{\to }\text{PMCA} \end{array}$$

Among these mechanisms there were two parameters that required calibration. First, we needed to tune the overall rate of calcium extrusion due to PMCA. While we had the parameters for its binding kinetics, we needed to tune the overall flux by altering the mechanism's surface density. Second, we had to alter the conductances of the VGCCs. In altering the model we had expanded the volume the model tracked while calculating calcium concentration. The original conductance values were tuned assuming a thin shell on the inner surface of the cell membrane. This expanded volume required increasing the channel conductances such that the calcium influx was enough to drive the fluctuations seen in the target experimental data.

As our target for constraining the intracellular calcium dynamics we chose the calcium fluorescence following an action potential (AP) (Power and Sah, 2002) as this protocol minimized the amount of calcium released from the ER. These experiments measured fluorescence transients in both the soma and the apical dendritic trunk, allowing us to calibrate separate parameter values for different section types. To calibrate these mechanisms we induced a simulated AP. We then modified the parameters values by hand until the simulated calcium fluorescence matched the target data. By altering the VGCC conductances we could alter the overall amplitude of the calcium transient. Due to differences in target amplitude, separate VGCC conductance values were calibrated for the soma and apical dendritic trunk. Increasing the density of PMCA decreased the maximum amplitude of the calcium along with increasing the rate the transient decayed to resting concentrations. The results of this calibration can be seen in Figure 2C.

### 2.3. Calibrating Calcium Release and Spike Acceleration

With the components for the calcium dynamics in place, the next step was to calibrate the production of IP_3_ following M1 mAChR activation. For the M1 mAChR model we turned to the kinetic models developed by the Hille lab (Falkenburger et al., 2010a,b, 2013; Kruse et al., 2016). This model included mechanisms that describe the process from the receptor activation by its agonist to PIP_2_ hydrolysis into IP_3_ and DAG. A schematic representation of this model including all of the associated reactions can be found in Figure S2. However, the model required notable modifications to fit our purpose. First, the Hille model simulated the agonist Oxo-M, not acetylcholine (ACh). While Oxo-M is an important muscarinic agonist, the goal of simulating endogenous cholinergic modulation required the mechanism to include ACh. Our work added the action of ACh on the M1 mAChR model through the calibration of additional parameters. Second, the rate of IP_3_ production was extremely slow compared to the behavior seen in CA1 pyramidal cells. Recordings of spiking CA1 pyramidal cells exposed to brief (40 ms) pulses of ACh were provided by the authors of Gulledge and Kawaguchi (2007). From these recordings we selected a subset of traces demonstrating regular spiking activity where the pre-ACh spiking frequency was <15 Hz. This provided 16 cell voltage traces. From these selected recordings it was determined that the regenerative release occurred within 200 ms of receptor activation as spikes were inhibited by this time. Both of these issues required the alteration of parameter values in order to achieve the desired responses.

In Falkenburger et al. (2010b), the authors used fluorescence resonance energy transfer (FRET) to measure the binding of M1 mAChRs to Oxo-M. A separate study performed an analysis of ACh binding to M1 using similar FRET techniques (Ziegler et al., 2011). From this study we took the half maximal effective concentration value of ACh and used that value to calibrate the parameters for agonist binding the receptor (see reaction 1 in Table S2). The change in the receptor's response to agonist concentration can be seen in Figure S3.

The discrepancy between the rapid release of intracellular calcium after M1 activation seen in CA1 pyramidal cells and the slow generation of IP_3_ in the Kruse et al. (2016) model was solved by increasing a subset of kinetic parameters in two portions of the M1 model. This discrepancy is most likely due to the original model being constrained to fit the response within sympathetic neurons. Activation of M1 channels in this neuron type leads to PIP_2_ depletion but not large releases of intracellular calcium. The rate of PLC activity will differ depending on the specific isozymes present within the cell type. Hippocampal cells contain PLC isozymes which are activated by increased intracellular calcium (Nakahara et al., 2005), creating a positive feedback for the hydrolysis of PIP_2_. It stands to reason that PIP_2_ hydrolysis would be triggered more rapidly than in the original model. The first portion of the model that needed faster dynamics was the activation and inactivation of PLC through its binding and unbinding to the G protein. Expressions (10) and (11) describe these reactions.

(10) $$\begin{array}{l} \text{PLC}+G\alpha -\text{GTP}\overset{k^{PLCassoc}}{\to }G\alpha -\text{GTP-PLC} \end{array}$$

(11) $$\begin{array}{l} G\alpha -\text{GDP-PLC}\overset{k^{PLCdiss}}{\to }G\alpha -\text{GDP}+\text{PLC} \end{array}$$

**Figure 3:**
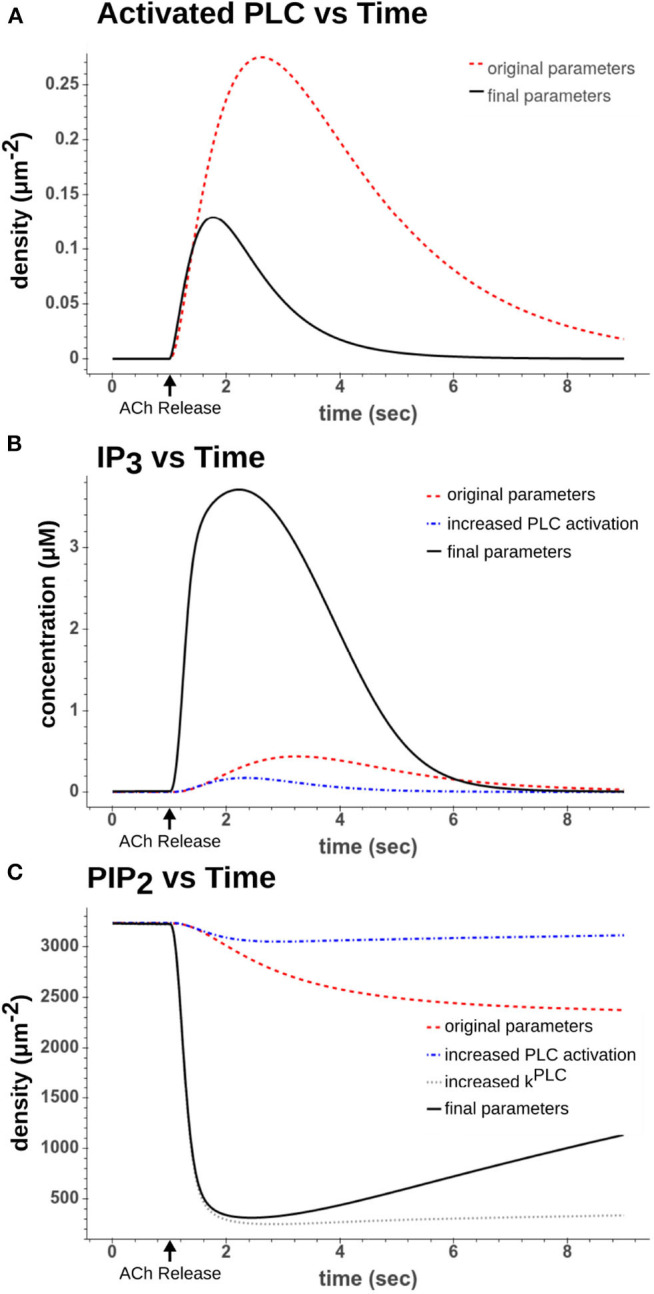
Comparison of dynamics after a simulated 50 ms 100 μM ACh pulse. **(A)** Activated PLC dynamics using original parameter values from Kruse et al. (2016) (red dash) to recalibrated parameter values used in final model (black solid). **(B)** IP_3_ dynamics using either the original parameters (red dash), increased PLC activation parameters (blue dash-dot), or increased PLC activation parameters along with increased hydrolysis rate (*k*^*PLC*^) (black solid) **(C)** PIP_2_ dynamics using either using either the original parameters (red dash), increased PLC activation parameters (blue dash-dot), increased PLC activation parameters along with increased hydrolysis rate (*k*^*PLC*^) (gray dot), or all increased parameters including those that drive synthesis of PIP_2_ (black solid).

**Figure 4:**
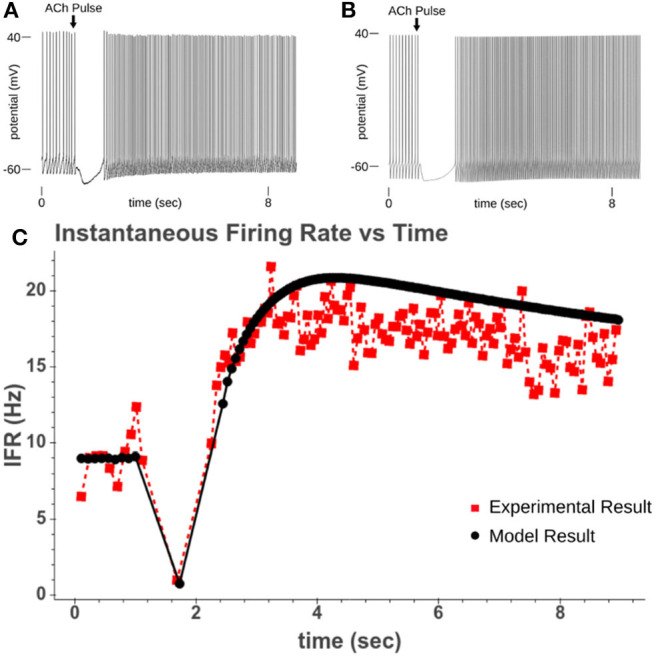
**(A)** Experimental recording of CA1 pyramidal cell responding to a 40 ms pulse of 100 μM while driven to spiking. **(B)** Model response to 50 ms pulse of 100 μM ACh while cell is driven to regular spiking. **(C)** Comparison of instantaneous firing rate of experimental response and model response.

From these reactions we recalibrated the two forward rates (*k*^*PLCassoc*^ and *k*^*PLCdiss*^). If we examine the original dynamics as seen in Figures 3A,B, one can see that the PLC activation peaks around 2 s after the ACh pulse and that IP_3_ levels peak around the same time. However, looking at the cell recordings (see Figure 4A for an example), by this time the calcium transients have already largely ended by 2 s as the cells have largely resumed spiking by then. Using the original parameter values led to a longer weak release of calcium from the ER as opposed to the approximately 1 s duration strong release we required to reach higher (>1 μM) intracellular calcium concentrations. The two parameters were therefore both increased by a factor of 10. The difference in dynamics can be seen in Figure 3A.

The second portion of the M1 model that required changed kinetics was the hydrolysis of PIP_2_ into DAG and IP_3_. Expressions (12) and (13) describe this reaction.

(12) $$\begin{array}{ll} \text{PIP2} & \overset{k^{PLC}}{\to }{\text{IP}}_{3}+\text{DAG} \end{array}$$

(13) $$\begin{array}{ll} k^{PLC} & =r_{PLC}*G\alpha -GTP-PLC \end{array}$$

Here the parameter, *r*_*PLC*_, was increased by a factor of 100. If we look at Figure 3B, we can see how this altered the dynamics of the reaction. By increasing the rate of the hydrolysis along with increasing the rates in Expressions (10) and (11), the production of IP_3_ occurred far more rapidly and was largely complete within 2 s of the ACh pulse. This also rapidly depleted the PIP_2_ as seen in Figure 3C.

The next goals were to calibrate the calcium release required for spike inhibition and the rate of PIP_2_ synthesis. This latter process controls the rate of reactivation of the M-current, and thereby controls the duration of spike acceleration. With the rate of IP_3_ increased in previous calibration steps, overall IP_3_ levels were controlled by altering the total concentrations of IP_3_ Kinase (IP3K) (Expressions 16, 17, and 18) and IP 5-phosphatase (IP5P) (Expressions 14, 15). Similar reasoning to the increased rate of IP_3_ production drove tuning the rate of IP_3_ removal. The concentration of IP_3_ needed to return to near resting levels quickly enough that calcium release ended within seconds of the ACh pulse. This allowed mechanisms to restore calcium to the ER and also allowed the cell's activity to sharply transition from hyperpolarization to accelerated spiking.

(14) $$\begin{array}{ll} {\text{IP}}_{5}\text{P}+\text{IP3} & \overset{k_{f}^{ip5p}}{\underset{k_{b}^{ip5p}}{⇌}}{\text{IP}}_{5}\text{P}-{\text{IP}}_{3} \end{array}$$

(15) $$\begin{array}{ll} {\text{IP}}_{5}\text{P}-{\text{IP}}_{3} & \overset{k^{ip2}}{\to }{\text{IP}}_{5}\text{P}+{\text{IP}}_{2} \end{array}$$

(16) $$\begin{array}{ll} {\text{IP}}_{3}\text{K}+2{\text{Ca}}^{2+} & \overset{k_{f}^{ip3k\;ca}}{\underset{k_{b}^{ip3k\;ca}}{⇌}}{\text{IP}}_{3}\text{K}-2{\text{Ca}}_{2} \end{array}$$

(17) $$\begin{array}{ll} {\text{IP}}_{3}\text{K}-{\text{Ca}}_{2}+{\text{IP}}_{3} & \overset{k_{f}^{ip3k\;ip3}}{\underset{k_{b}^{ip3k\;ip3}}{⇌}}{\text{IP}}_{3}\text{K}-2{\text{Ca}}_{2}-{\text{IP}}_{3} \end{array}$$

(18) $$\begin{array}{ll} {\text{IP}}_{3}\text{K}-{\text{Ca}}_{2}-{\text{IP}}_{3} & \overset{k^{ip4}}{\to }{\text{IP}}_{3}\text{K}-{\text{Ca}}_{2}+{\text{IP}}_{4} \end{array}$$

The mechanism for calcium efflux through IP_3_ receptors is described by Expressions (19) and (20). Here *R*_*Open*_ refers to the open state of the full kinetic model. For the full kinetic scheme of the IP_3_ receptor model (see Figure S2B).

(19) $$\begin{array}{l} Ca_{ER}^{2+}\overset{k_{f}^{IP3R}}{\to }Ca_{cyt}^{2+} \end{array}$$

(20) $$\begin{array}{l} k_{f}^{IP3R}=g_{IP3R}\cdot R_{Open} \end{array}$$

By manipulating the maximum flux, *g*_*IP*3*R*_, and the rate of IP_3_ breakdown we were able to produce calcium transients with peaks reached >1 μM that resolved within the desired duration range (1–3 s).

The final set of parameters we altered to replicate the CA1 pyramidal cells' behavior were involved in the synthesis of PIP_2_. The resynthesis of PIP_2_ is due to activity in the ER that varies with the proteins a cell type expresses (Blunsom and Cockcroft, 2020). As these species are not characterized within the CA1 pyramidal cell, we chose to use the mechanisms present in the model and to calibrate the kinetic parameters to match the behavior seen in *in vitro* experiments. The following expressions describe these reactions.

(21) $$\begin{array}{ll} \text{PI} & \overset{k^{4K}}{\to }\text{PI(4)P} \end{array}$$

(22) $$\begin{array}{ll} \text{PI(4)P} & \overset{k^{5K}}{\to }{\text{PIP}}_{2} \end{array}$$

(23) $$\begin{array}{ll} \text{PI(4)P} & \overset{k^{4P}}{\to }\text{PI} \end{array}$$

(24) $$\begin{array}{ll} {\text{PIP}}_{2} & \overset{k^{5P}}{\to }\text{PI(4)P} \end{array}$$

From these expressions four parameters needed to be recalibrated (*k*^4*K*^, *k*^5*K*^, *k*^4*P*^, and *k*^5*P*^). These parameters were recalibrated based upon the rate the instantaneous firing rate (IFR) returned to its pre-ACh value. In our model, this increased spiking is due to the inhibition of the M-current following PIP_2_ depletion. By simulating the original experiments used in Gulledge and Kawaguchi (2007), we could replicate the altered spiking behavior and calibrate the kinetic parameters for PIP_2_ synthesis so that the resolution of spike acceleration matched the experimental results. Figure 3C shows how the recalibrated parameters changed the synthesis of PIP_2_. Figure 4 shows a simulated experiment along with an example of a cell recording and demonstrates the model's ability to replicate the changes to IFR over time.

### 2.4. Depolarization Activated Calcium Store Replenishment

The final mechanism we resolved to include in this model was the role of store operated calcium entry (SOCE). Briefly, this is a process by which the depletion of lumenal calcium causes the activation of calcium channels on the plasma membrane. These channels are positioned in membrane junctions or regions where the distance between the plasma and ER membrane is <100 nm. This allows the calcium that enters through SOCE to almost directly enter the ER without altering the overall intracellular calcium concentration. For a more in depth review of this process (see Majewski and Kuznicki, 2014). Interestingly this process is also dependent on depolarization of the cell (Dasari et al., 2017). Without depolarization, repeated phasic exposure fails to demonstrate repeated hyperpolarizing responses to intracellular calcium release.

While this process is well documented and the responsible actors have been partly identified, the kinetics of this process have not been quantified. Not including a mechanism to replicate SOCE would make it impossible to simulate network activity with synaptic release of ACh, as the cell model would only be able to respond to one release event. To overcome this limitation we included a mechanism that replicated the behavior of SOCE without explicitly modeling the underlying molecular events. This mechanism is based on a series of assumptions.

- Depolarization is required for activation (Dasari et al., 2017).
- Hyperpolarization does not cause a leak from intracellular stores.
- *Ca*_*ER*_ depletion is required for activation (Majewski and Kuznicki, 2014).
- The action of this mechanism bypasses the intracellular calcium concentration as calcium directly moves from the extracellular space to the ER lumen.

Expression (25) was the mechanism we used which fit the above criteria.

(25) $$\begin{array}{l} dCa_{ER}^{2+}=g_{SOCE}\cdot \ln\left(1+e^{v_{m}-v_{init}}\right)\cdot e^{\frac{-\left(Ca_{ER}-Ca_{d}\right)}{k_{SOCE}}} \end{array}$$

This mechanism avoids altering the intracellular calcium concentration by directly changing the value of *Ca*_*ER*_. Through the use of a softplus function this mechanism will have minimum activation except when the cell's membrane potential is depolarized from it's resting value (*v*_*init*_). Also as *Ca*_*ER*_ approaches its resting value, this mechanism deactivates, ensuring it is maximally activated after calcium store depletion. As seen in Figure S4 this mechanism allows repeated hyperpolarizations following intracellular calcium release if the cell depolarizes, but intracellular calcium release cannot repeatedly occur if the cell maintains a near resting membrane potential. This replicates behavior seen in cortical pyramidal cells (Dasari et al., 2017).

## 3. Results

### 3.1. Acetylcholine and Cell Excitability

With the compartmental model able to replicate experimental responses of CA1 cells, we sought to explore how variations in the concentration of ACh would alter the model's behavior. As our model only captures the modulation in the soma, axon, and apical trunk of the cell, we focused on simulating how ACh alters the cell's excitability. Experiments tend to use agonist concentrations that will drive a significant and unambiguous response. These experimental concentrations may not be biologically relevant, however. While some measurements of *in vivo* ACh concentrations have been made, these measurements were made under the assumption of volumetric transmission. Recent work, however, has demonstrated that cholinergic terminals form synapses, undermining this volumetric assumption (Takács et al., 2018). It therefore remains unclear what concentrations muscarinic receptors see during cognition. This particular aspect will be further discussed later. To avoid testing an exact concentration profile we sought to explore how our model responds to a wide range of concentrations. We also explored how the model behaved differently under a synaptic release of ACh vs. a steady state exposure. Short pulses (50 ms) replicated a simultaneous synaptic release we termed “phasic,” while long term (>5 s) exposures simulated a steady state exposure referred to as “tonic” exposure.

**Figure 5:**
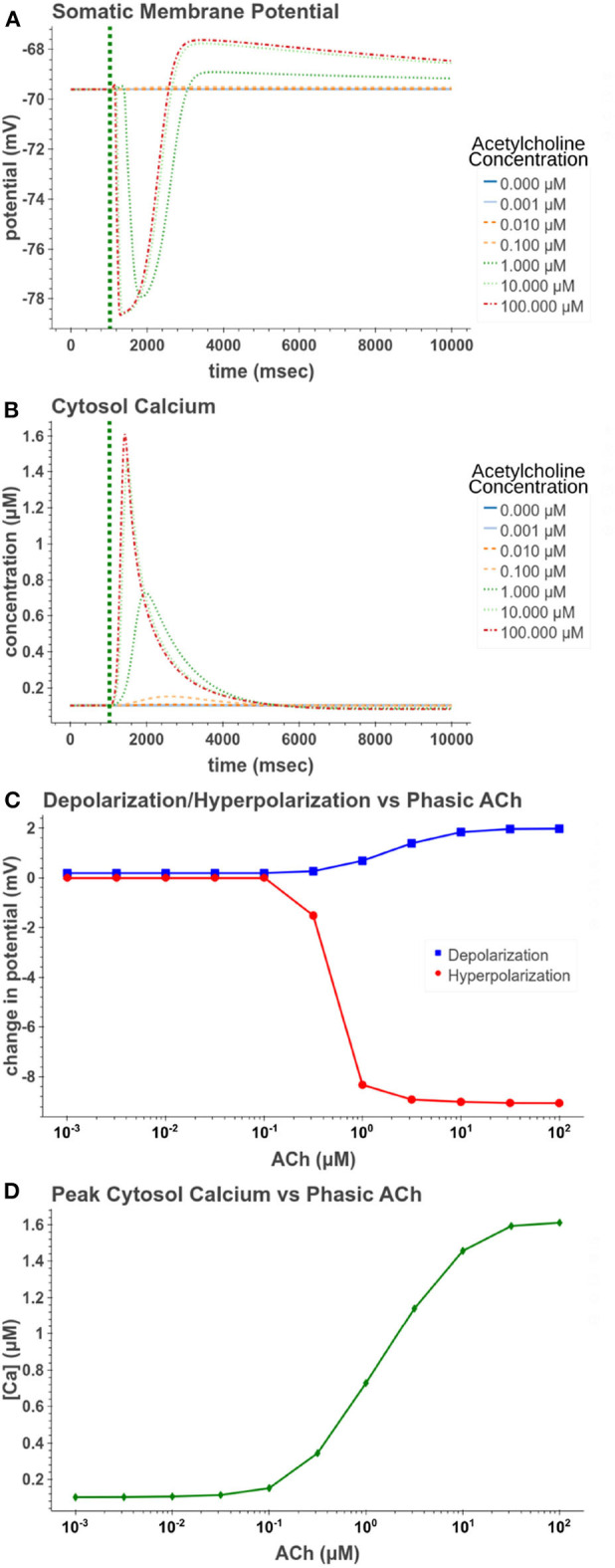
Simulated response to phasic (50 ms) exposure at varying concentrations of acetylcholine. **(A)** Cell membrane potential with no other stimulation besides acetylcholine pulse. **(B)** Simulated intracellular calcium release. **(C)** Peak hyperpolarization and depolarization values at different concentrations of acetylcholine. **(D)** The peak intracellular calcium concentration following acetylcholine pulse.

We first replicated phasic exposure while the cell is at a resting membrane potential. Figure 5 demonstrates how the model replicates these changes to membrane potential. At 100 μM the model produces a −9.05 mV hyperpolarization followed by a depolarization of 1.98 mV. From Figures 5C,D, it is clear that the amplitudes of these reactions are highly concentration dependent. From Figure 5D, its clear that for concentrations <0.1 μM, the release of calcium from intracellular stores is negligible and consequently no hyperpolarization occurs. In Figure 5C, we see that the hyperpolarizing effect is induced at lower concentrations (EC_50_ = 0.499 μM) than the depolarization (EC_50_ = 1.95 μM). This suggests that for cells at rest, within a certain range of concentrations, short pulses of acetylcholine would only have an inhibitory effect on the cell without producing much excitatory modulation.

**Figure 6:**
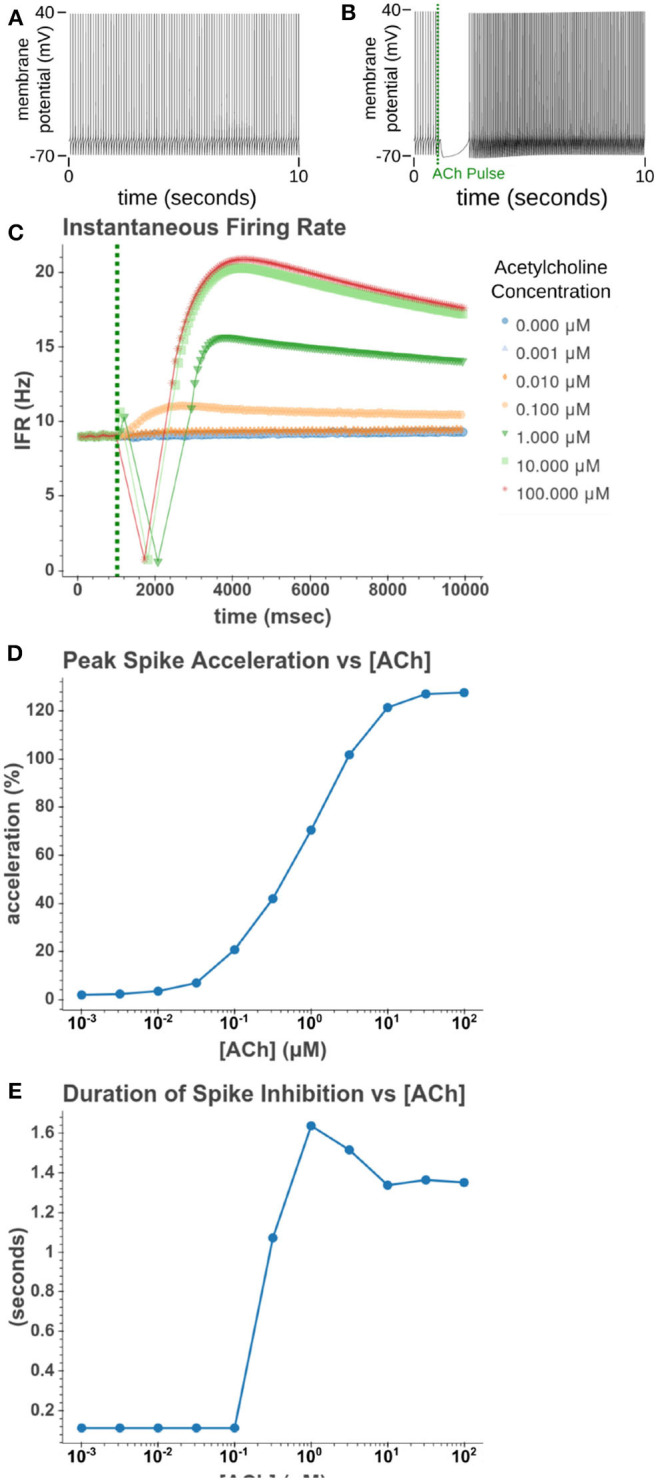
Simulated response to phasic (50 ms) exposure of varying concentrations of acetylcholine. **(A)** Cell injected with a current amplitude such that it spikes at a steady rate of 10 Hz. Current injection is present throughout simulation. **(B)** Repeat of experiment with the addition of a 100 μM acetylcholine pulse that starts at *t* = 1 s and lasts for the duration of the simulation. **(C)** Instantaneous firing rate over time for different concentrations of ACh. This rate is the inverse of the inter spike interval. **(D)** The peak spike acceleration increased with higher concentrations of ACh. **(E)** The duration of the pause in spiking vs. the concentration of ACh.

**Figure 7:**
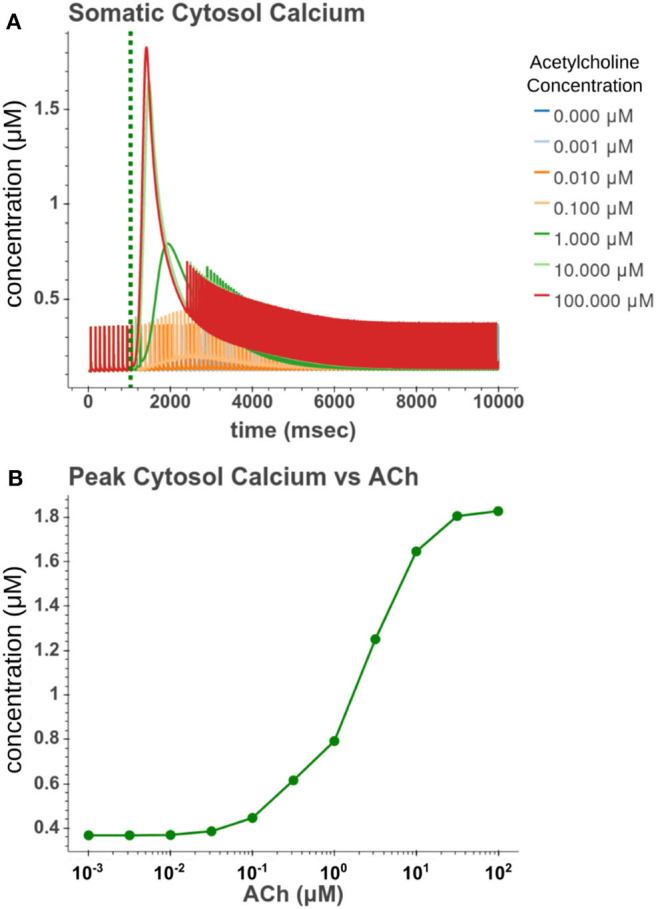
**(A)** Time series of intracellular calcium concentration after phasic (50 ms) exposure to ACh. **(B)** Peak cytosol calcium vs. the concentration of the phasic ACh pulse.

We then sought to explore how phasic ACh exposure would alter the spiking activity of the cell model. We ran a series of simulations in which current injections drove the cell to spike at a constant rate of 10 Hz. At the 1 s mark we then modeled the injection of a 50 ms pulse of ACh with each simulation having a different concentration. The results of these simulations can be seen in Figure 6. Looking at Figure 6C, we can see that once spiking resumes, the IFR increases rapidly reaching a peak around 2–3 s after the ACh pulse. The increased firing rate then slowly returned to its baseline rate over several seconds. We calculated the peak percent increase in IFR, or spike acceleration, for each simulated concentration. As shown in Figure 6D, this value formed a smooth sigmoidal curve when plotted against concentration. The duration of the spike inhibition, seen in Figure 6E varied in a way similar to the variation seen in the resting conditions, abruptly beginning at concentrations above 0.1 μM. That spike inhibition begins so abruptly means that for ACh concentrations of 0.1 μM or less the firing rate will have noticeably increased without a period of spike inhibition. Additionally, while the peak acceleration followed a sigmoidal curve, the longest spike inhibition occurred at 1 μM, with the duration of inhibition decreasing thereafter. If we examine Figure 7, we can see the cause of this nonlinearity. In Figure 7A, we can see that as the concentration of the ACh pulse increases, the peak concentration of the intracellular calcium transient increases. Figure 7B demonstrates that these peak values form a sigmoidal curve. However, while the peak values is increasing, the duration of the calcium transient is also decreasing. This is due to larger ACh pulses driving increased IP_3_ production and thereby causing a more rapid depletion of ER calcium stores. As the stores are depleted, the calcium transient begins to decay. It is this accelerated depletion of ER calcium which leads to the shorter duration of inhibition for higher concentrations of ACh. This nonlinearity in spike inhibition could have interesting implications for network activity, and will be a subject of discussion later in this paper.

**Figure 8:**
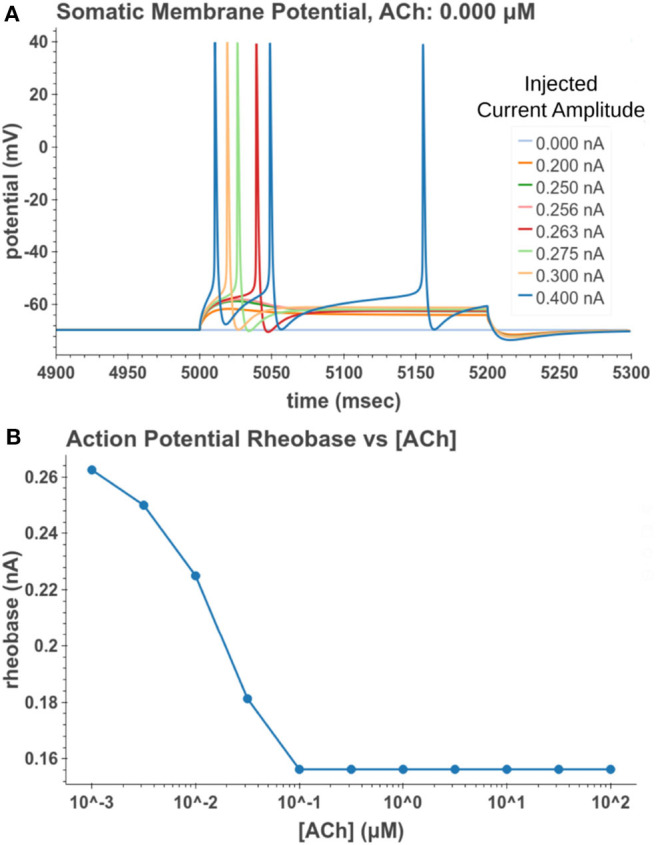
Measuring reduction in rheobase due to tonic acetylcholine exposure. **(A)** A 200 ms current pulse of varying amplitude is applied at a time sufficiently after the start of a simulated acetylcholine exposure such that the system is at steady state. A binary search was performed to find the minimum current injection amplitude which would generate an action potential. **(B)** Cell rheobase decreases with increased concentration of acetylcholine.

**Figure 9:**
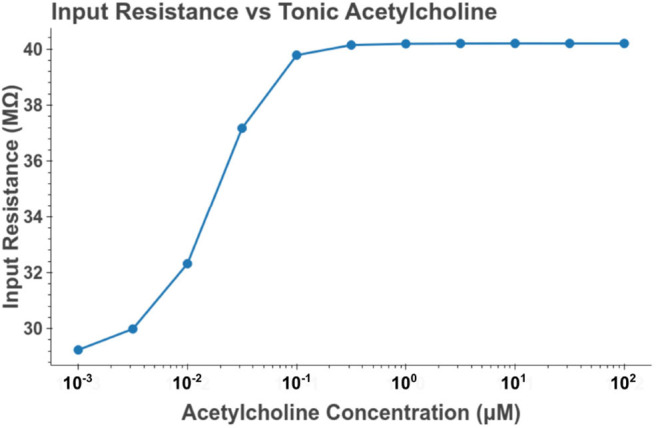
Increasing the concentration of tonic acetylcholine increases the input resistance of the cell model as measured at the soma. Input resistance was measured by performing a series of somatic current injections and then performing linear regression on the relation between membrane depolarization to current amplitude. The values plotted are the slopes of the estimated linear functions. The current amplitudes used were 0, −100, and 100 pA.

**Figure 10:**
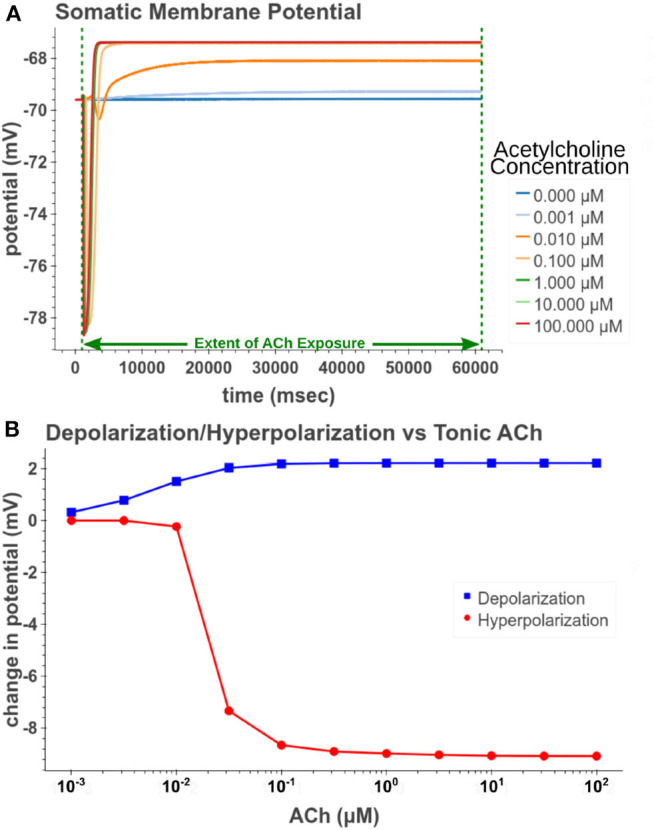
Measuring depolarization and hyperpolarization after tonic (60 s) exposure to acetylcholine. **(A)** Simulated response of somatic membrane potential to different concentrations. **(B)** Amplitude of steady depolarization and temporary hyperpolarization vs. acetylcholine concentration.

**Figure 11:**
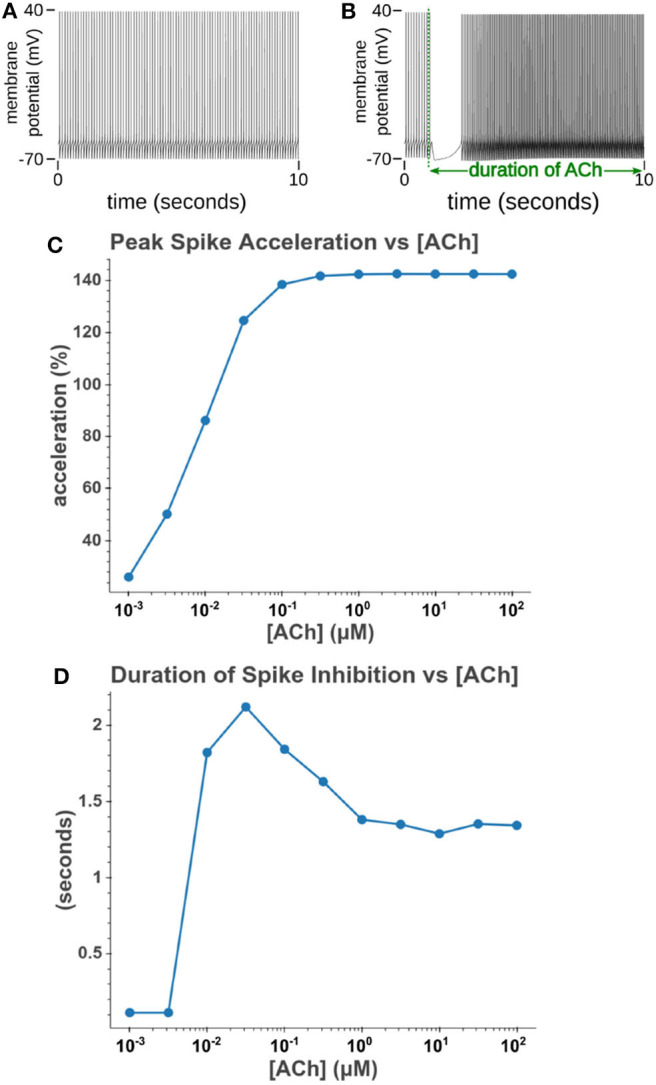
Increasing the concentration of tonic acetylcholine increases spike rate for a given injected current amplitude. **(A)** Cell injected with a current amplitude such that it spikes at a steady rate of 10 Hz. Current injection is present throughout simulation. **(B)** Repeat of experiment with the addition of a 100 μM acetylcholine pulse that starts at *t* = 1 s and lasts for the duration of the simulation. **(C)** The maximum spike frequency acceleration vs. acetylcholine concentration. Spike frequency acceleration was measured as the percent increase from the rate before acetylcholine exposure. **(D)** Duration of spike inhibition vs. tonic acetylcholine concentration as measured as the longest inter spike interval after the initiation of the acetylcholine pulse.

Under tonic exposure to ACh, we noted multiple ways that the cell model displayed increased excitability. As can be seen in Figure 8 the rheobase (defined here as the minimum amplitude of a 200 ms current pulse required to elicit an action potential) decreased with increasing concentrations of ACh. Starting at a value of 263 pA, the rheobase decreased 40.5% to a value of 156 pA with 14.3 nM of ACh producing half of the maximum decrease. The cell model also demonstrated increased excitability, illustrated by an increase of 39.1% in the input resistance measured at the soma. The increase in simulated input resistance can be see in Figure 9. This increased excitability plateaus in the high hundred nanomolar range with the increased excitability starting within nanomolar concentrations. This suggests that even relatively low background concentrations should be able to alter cell spiking behavior. Long term exposure of ACh also produced a depolarization that persisted through the duration of exposure. The amplitude of this depolarization varied as a function of ACh concentration as demonstrated in Figure 10. Finally, tonic exposure caused accelerated spiking for a given amplitude of current injected at the soma. This accelerated spiking is demonstrated in Figure 11.

### 3.2. Intracellular Calcium Release

**Figure 12:**
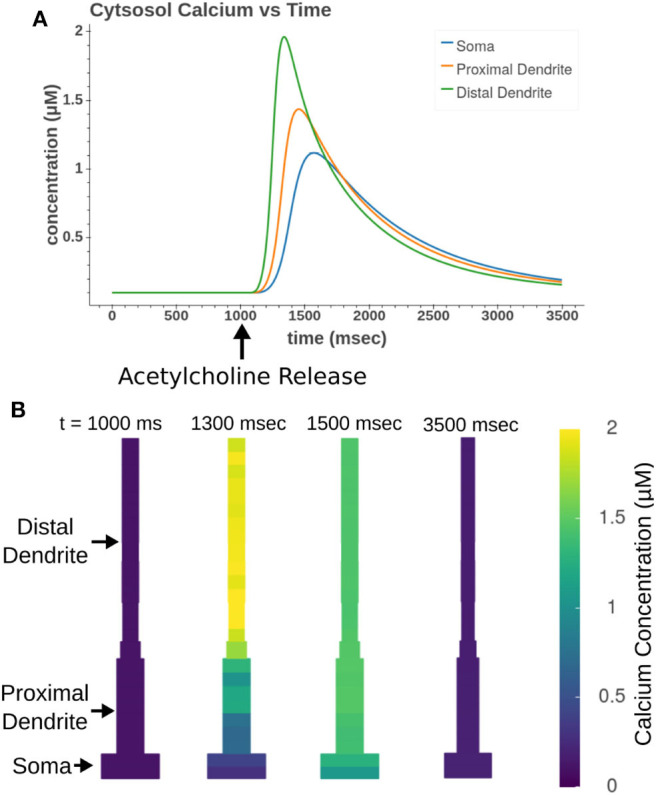
Acetylcholine leads to the release of intracellular calcium. All sections in the apical trunk and soma were simultaneously exposed to a 50 ms pulse of 100 μM ACh. **(A)** Simulated time course of intracellular calcium after acetylcholine exposure. More distal sections achieve higher concentrations more rapidly than somatic sections. **(B)** Display of concentrations in all model compartments at different time steps throughout calcium wave.

Focal application of muscarinic agonists and stimulation of cholinergic terminals were demonstrated to generate calcium waves that progressed from the apical dendritic trunk to the soma (Power and Sah, 2002). Our model, as demonstrated in Figure 12, replicates many aspects of these calcium waves. As seen experimentally, the sections in the apical trunk reached a higher peak calcium concentration more rapidly than the somatic section. This is likely due to dendritic regions having higher surface area to volume ratios.

## 4. Discussion

### 4.1. Novel Additions to CA1 Compartmental Model

This model includes a number of mechanisms that have largely been absent from previous compartmental computational models of the CA1 pyramidal cell. In addition to including the M1 mAChR model, the intracellular calcium related mechanisms have been greatly expanded. Among these new calcium mechanisms were calbindin and PMCA. We have also been able to replicate the calcium wave phenomenon by including the endoplasmic reticulum. The parameters for these mechanisms were calibrated using experimental measurements obtained in CA1 pyramidal cells to ensure the resulting model accurately replicates this cell type's behavior. The addition of these novel mechanisms allows our model to replicate several molecular interactions that have been heretofore ignored in whole cell computational models of CA1 pyramidal cells.

### 4.2. Predictions From Model

The expanded CA1 pyramidal cell model have allowed us to generate some predictions which could be tested experimentally. First, the model predicts that intracellular calcium release can be triggered over a wide range of ACh concentrations. Later experimental evidence may show a more tightly regulated threshold that the transition between minimum and maximum responses occurs over a narrower range of concentrations. These hypothetical results would then suggest that there are mechanisms involved which introduce additional nonlinearities that increase the threshold for calcium release. For example the rate of PIP_2_ hydrolysis into IP_3_ being dependent on calcium would likely cause a sharper threshold for calcium waves.

A second prediction is that the duration of spike suppression as seen in Figures 6E, 11D does not increase monotonically; instead the maximum duration occurs at intermediate concentrations (0.1 μM for tonic and 1 μM for phasic exposure). This result is likely due to the interplay between two competing processes: regenerative calcium release and the rate of calcium store depletion. The concentrations with the longest suppressions generate enough IP_3_ to drive regenerative calcium release through IP_3_Rs while minimizing the rate of Ca^2+^ release from intracellular stores. Higher ACh concentrations drive higher IP_3_ production and so IP_3_Rs open more fully and deplete intracellular stores more rapidly. This modulation of the length of inhibition is interesting when considering the possible functional roles calcium waves play in CA1 pyramidal cells.

If the function of calcium waves is to provide an inhibitory signal, then this inhibitory signal would have some interesting properties. First, as IP_3_ is the trigger for this inhibition, multiple sources (whether mGluRs or mAChRs) could be required to work in concert to generate this signal. The non-monotonically increasing duration of spike cessation suggests that coactivation of additional IP_3_ sources after the regenerative calcium release threshold has been passed may cause a shorter inhibition as the additional IP_3_ will only lead to faster calcium stores depletion. Second, the rate at which intracellular calcium stores are depleted depends upon the amount of calcium stored. A cell with more calcium buffered in the ER will have a longer inhibitory reaction to cholinergic modulation. Since every action potential increases the amount of calcium in the ER, calcium waves would be longer for cells that have had more action potentials in the recent past. This mechanism would thereby act as an internal inhibition which encodes each cell's past activity. These two properties suggest scenarios where cholinergic modulation causes shorter inhibition for cells that are currently receiving a large glutamatergic signal but have not been spiking much in the past while cells that have been consistently spiking and only receive cholinergic modulation are inhibited for a longer period. The implications of these properties may have a crucial impact on the downstream effects of calcium homeostasis, amongst which are excitotoxicity and learning and memory; these aspects will be studied in later work (see section 4.4 below).

### 4.3. Refining Model

In developing this model there have been gaps in experimental evidence which have made it difficult to model all of the experimental reactions to cholinergic modulation. First of all the dynamics of the phosphoinositides in the CA1 pyramidal cell plasma membranes are not well understood. This has forced us to make assumptions based on electrophysiological results, but further research into this area would aid in refining the model. As the ER plays a role in the production of these phospholipids, it is likely that the depletion of calcium stores leads to changed dynamics. Indeed experiments in CA1 pyramidal cells have suggested that prolonged activation of mAChRs can drive oscillations in PIP_2_ levels (Hackelberg and Oliver, 2018). The signaling cascade that drives these oscillations, however, is not well-understood and therefore could not be included in this model iteration. As these dynamics become better understood, more explicit cascades can be incorporated into the model allowing for a better simulation of the depletion and synthesis of PIP_2_.

Tonic cholinergic activation was also shown to inhibit the early portions of slow after hyperpolarization (sAHP) following trains of action potentials (Dasari and Gulledge, 2011). Experimental evidence suggests this sAHP is largely due to sodium-potassium exchange pumps (Gulledge et al., 2013; Tiwari et al., 2018). It is unclear how these exchange pumps interact with the mechanisms involved with mAChR activation. Without this clearer understanding, we have no way to properly calibrate the level of sAHP inhibition to variations in ACh concentration.

The model could also be expanded through adding mechanisms which model mitochondrial calcium dynamics. The mitochondria, along with being vital for the energy metabolism of the cell, play a large role in calcium dynamics through interactions with the ER (Krols et al., 2016). As mitochondrial dysfunction has a well established link with Alzheimer's disease (Cenini and Voos, 2019), this expansion would provide a method for exploring the functional consequences to network behavior and how best to intercede.

### 4.4. Future Uses

Though the present work is a significant advancement for modeling the interactions between cholinergic input, intracellular calcium, and neuronal dynamics, the model is far from encompassing all of the mechanisms that participate in cholinergic response. Yet this work represents a framework within which additional mechanisms can be added as the knowledge of the system evolves. We have sought to use best practices while generating the code base to facilitate its understanding and allow future users to expand upon its capabilities. The current cell model focused on cholinergic modulation in the apical dendritic trunk and the somatic region and consequently does not incorporate the modulation of synaptic transmission. Experimental evidence has shown that in synapses originating from the CA3 region, the activation of presynaptic M4 mAChRs suppresses the amplitude of excitatory postsynaptic potentials (EPSPs) (Dasari and Gulledge, 2011). This signal suppression has been suggested to shift control of CA1 pyramidal cell activity away from the CA3 toward synaptic inputs from the entorhinal cortex (EC). This is theorized to set the CA1 network into a state more conducive for encoding the sensory information encoded by the EC synapses (Hasselmo and McGaughy, 2004). However, the synaptic connections from the EC are located in the most distal portions of the CA1 pyramidal cell dendritic tree. In order for these inputs to become dominant, the CA1 pyramidal cell would need to become more sensitive to distal inputs. Our model has demonstrated that it is capable of replicating an increased excitability as measured by increased input resistance and lower rheobase at higher concentrations of ACh. This increased excitability, in conjunction with suppressed CA3 synaptic activity would replicate increased sensitivity to distal inputs. Our model thereby constitutes a solid foundation for future work exploring the consequences of this modulation for the integration of inputs from EC vs. CA3.

Additionally, synaptic connections from CA3 pyramidal cells to CA1 pyramidal cells demonstrate plasticity that is dependent upon postsynaptic calcium concentrations (Cummings et al., 1996). M1 mAChRs are known to be present at these synapses (Yamasaki et al., 2010) and have been linked to both long-term potentiation and long-term depression (Dennis et al., 2016). Our lab has already developed a kinetic model for the postsynaptic calcium seen in the spine head (Hu et al., 2018). While this previous model did not include any mechanisms to link M1 activation to intracellular calcium release, these missing mechanisms could easily be added. This would allow us to expand upon previous modeling efforts that sought to tie calcium dynamics to plasticity (Shouval et al., 2002) to explore how cholinergic modulation alters the network dynamics through long term changes in connectivity.

Another direction of interest would consist of expanding the model to explicitly model cholinergic synapses. Currently our simulations treat acetylcholine concentration as a fixed value which we change in a step-wise manner. The addition of cholinergic synapses would allow us to explore how the model responds to varying synaptic parameters. For example, tonic ACh concentration is based upon both the amount of ACh released but also the rate of hydrolysis due to acetylcholinesterase (AChE). This enzyme is the target for a class of drugs, AChE inhibitors, used in the treatment of AD. Exploring how these drugs alter CA1 network dynamics could point to better treatment strategies. Additionally, cholinergic synapses have been shown to cotransmit ACh with GABA (Granger et al., 2016; Takács et al., 2018). This cotransmission could have dramatic effects on network coherence.

Finally, although pyramidal cells are the most numerous cell type in the CA1, they are not alone. There are a variety of interneuron cell types which are also the subject of cholinergic modulation. If ACh does play a role in shifting the focus of information processing from synapses from the CA3 to synapses from the EC, interneurons likely contribute to this process. This is due to certain interneurons' ability to disinhibit CA1 pyramidal cells (e.g., CCK+ Basket Cells; Karson et al., 2009). Furthermore, interneurons also participate in the generation of network oscillations which help organize network processes (e.g., OLM cells; Mikulovic et al., 2018). Understanding how these cells, through ACh modulation regulate the overall network activity would aid our understanding of the complex role ACh plays in the hippocampus.

## Data Availability Statement

Publicly available datasets were analyzed in this study. This data can be found here: https://github.com/armerg/ca1_muscarinic_modulation/.

## Author Contributions

AM and J-MB: concept and design of study, analysis and/or interpretation of data, and drafting of the manuscript. AM: data acquisition. AM, J-MB, GY, and TB: critical revision and approval of the manuscript to be published. All authors contributed to the article and approved the submitted version.

## Conflict of Interest

The authors declare that the research was conducted in the absence of any commercial or financial relationships that could be construed as a potential conflict of interest.
