# Supplementary material for: A Computational Model of the Cholinergic Modulation of CA1 Pyramidal Cell Activity
Source: Front Comput Neurosci. 2020 Sep 4;14:75. doi: 10.3389/fncom.2020.00075 (PMC7509450; doi:10.3389/fncom.2020.00075)
Supplement: Supplementary file 1 [file Data_Sheet_1.PDF]

# Supplementary Material

## 1 FIGURES

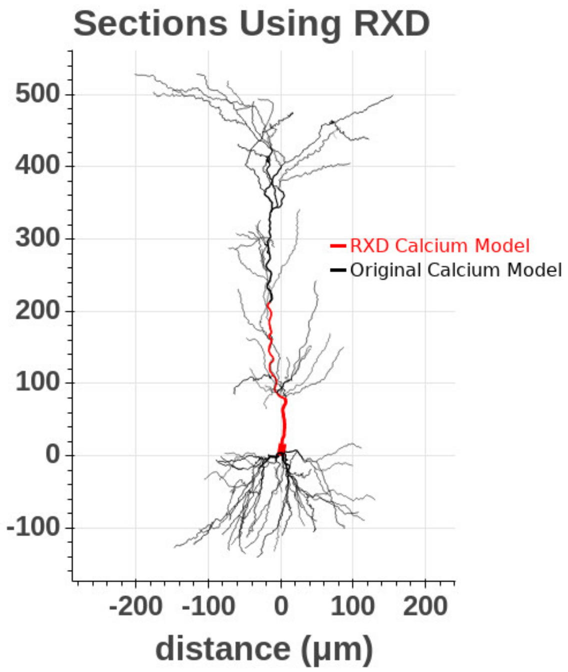


**Figure S1.** Visualization of compartmental model with expended calcium dynamics.

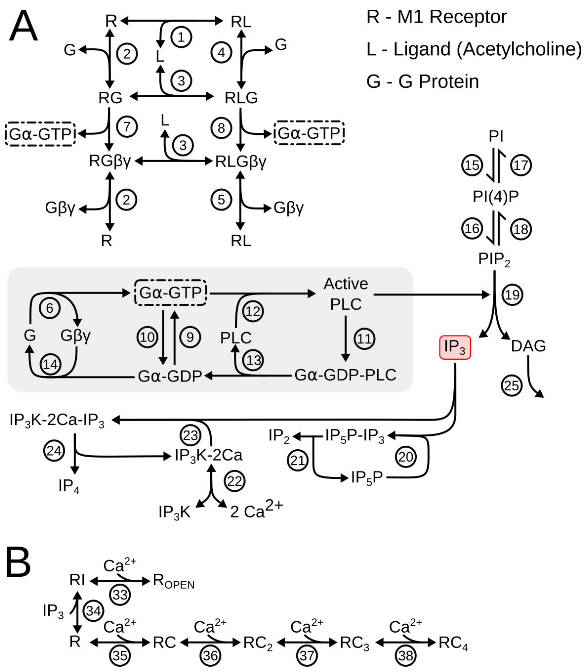


**Figure S2.** **A** Schematic representation of M1 receptor model including the mechanisms for the G protein cascade, $PIP_2$ synthesis, and $IP_3$ breakdown. The number next to each reaction is the entry in Table 2 in which the reaction kinetic parameters can be found. **B** Schematic representation of $IP_3R$ model used in model.

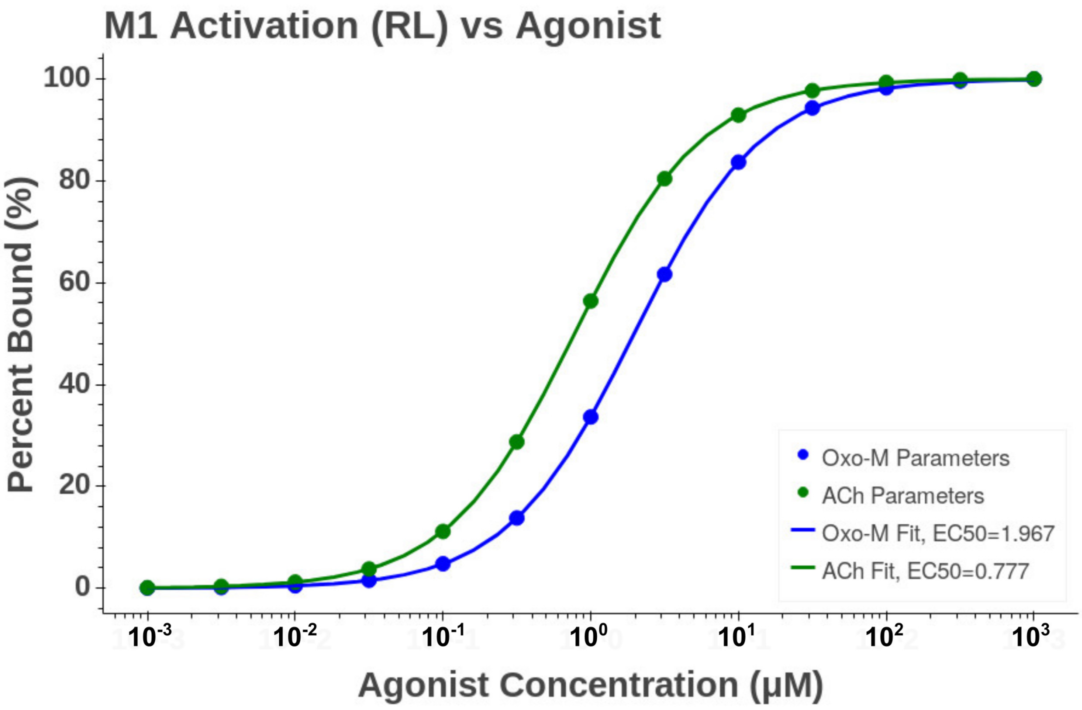


**Figure S3.** Comparison of concentration responses with original oxotremorine-m parameters in blue and new acetylcholine responses in green.

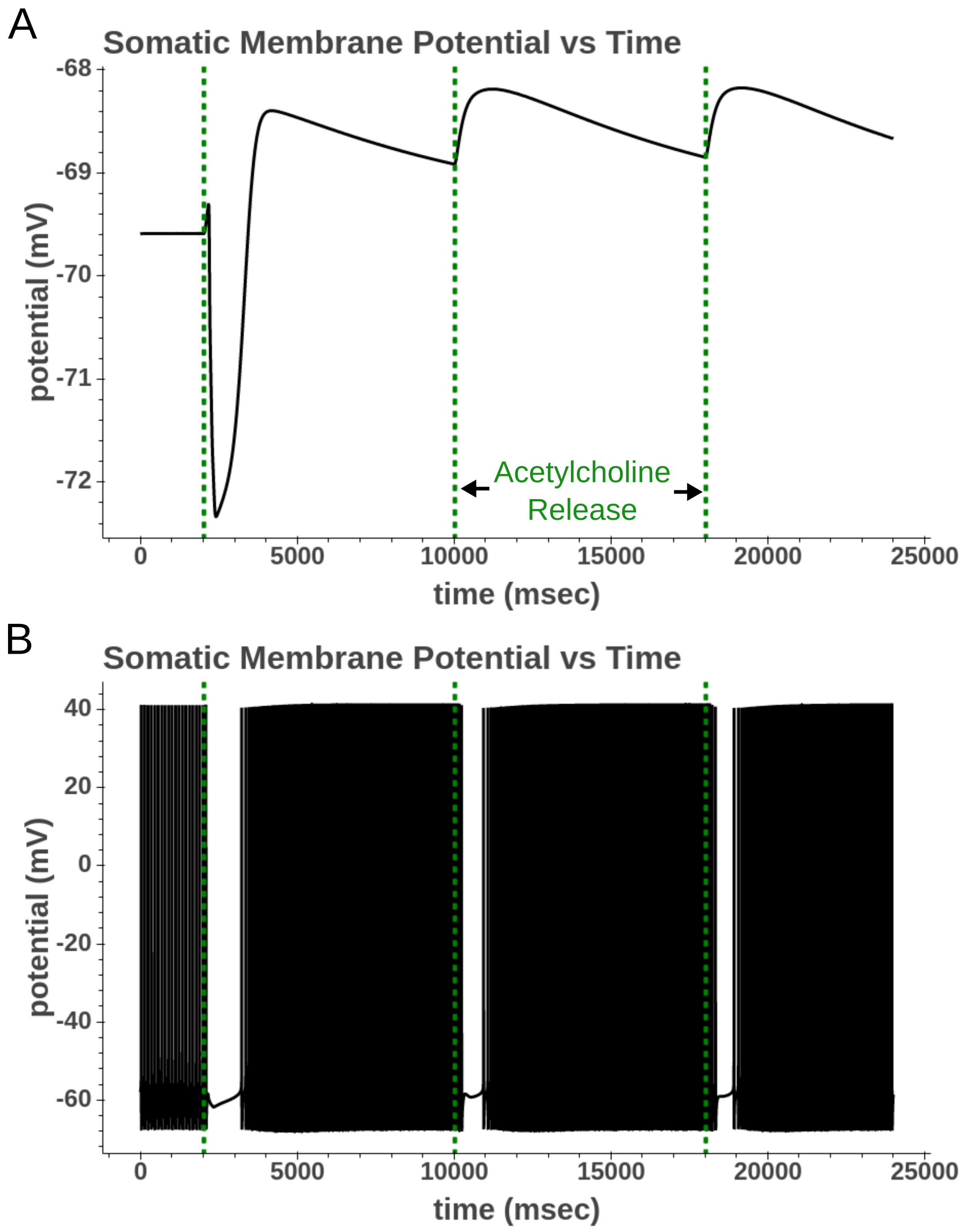


**Figure S4.** The inclusion of the SOCE mechanism allows for the rapid refilling of lumenal calcium if the cell is depolarized. **A** A cell unable to repeatedly release intracellular calcium if it remains at near resting membrane potentials, but **B** can perform repeated release if driven to spike.

## 2 TABLES

| Species | Initial Concentration | Model Region | Source |
|---|---|---|---|
| Calcium | 2.0 mM | Extracellular | (Doi et al., 2005) |
| PMCA | $2.56e^{-5}$ mM | Plasma Membrane | Tuned Value |
| $IP_5P$ | $2.5e^{-4}$ mM | Plasma Membrane | Tuned Value |
| $IP_3K$ | $1.0e^{-3}$ mM | Plasma Membrane | Tuned Value |
| M1 Receptor | 79.35 $\mu m^{-2}$ | Plasma Membrane | (Kruse et al., 2016) |
| G Protein | 40.0 $\mu m^{-2}$ | Plasma Membrane | (Kruse et al., 2016) |
| PLC | 15.6 $\mu m^{-2}$ | Plasma Membrane | (Kruse et al., 2016) |
| $PIP_2$ | 3232.0 $\mu m^{-2\,\dagger}$ | Plasma Membrane | (Kruse et al., 2016) |
| Bound $PIP_2$ | 6464.0 $\mu m^{-2\,\dagger}$ | Plasma Membrane | (Kruse et al., 2016) |
| PI(4)P | 4540.0 $\mu m^{-2\,\dagger}$ | Plasma Membrane | (Kruse et al., 2016) |
| PI | 226975.0 $\mu m^{-2\,\dagger}$ | Plasma Membrane | (Kruse et al., 2016) |
| DAG | 13.0 $\mu m^{-2}$ | Plasma Membrane | (Kruse et al., 2016) |
| Calcium | 100.0 nM | Intracellular | (Oh et al., 2013) |
| Calbindin-D28k | 45.0 $\mu$M | Intracellular | (Müller et al., 2005) |
| Oregon Green BAPTA-1 | 50.0 $\mu M^*$ | Intracellular | (Power and Sah, 2002) |
| $IP_3$ | 3.0 nM | Intracellular | Tuned Value |
| Calcium | 175.0 $\mu$M | Endoplasmic Reticulum | (Solovyova et al., 2002) |
| Calreticulin | 86.0 mM | Endoplasmic Reticulum | (Doi et al., 2005) |

**Table S1.** Table of mechanism initial concentrations. † initial value varies with the ratio of section volume to section surface area. * value is nonzero only for experiments requiring a simulated fluorescence signal.

| # | Reaction | Formula | Parameter Values | Source |
|---|---|---|---|---|
| 1 | ACh Binding to M1 R | $\text{ACh} + \text{R} \xrightleftharpoons[k_b^{L1}]{k_f^{L1}} \text{RL}$ | $k_f^{L1} = 2.78\ mM^{-1}\ ms^{-1}$<br>$k_b^{L1} = 2.15 \cdot 10^{-3}\ ms^{-1}$ | Alterred from (Falkenburger et al., 2010) |
| 2 | M1 R Binding to G Protein | $\text{R} + \text{G} \xrightleftharpoons[k_b^{G1}]{k_f^{G1}} \text{RG}$ | $k_f^{G1} = 6.8 \cdot 10^{-3}\ \mu m^2\ ms^{-1}$<br>$k_b^{G1} = 6.8 \cdot 10^{-3}\ ms^{-1}$ | Alterred from (Falkenburger et al., 2010) |
| 3 | ACh Binding to RG | $\text{ACh} + \text{RG} \xrightleftharpoons[k_b^{L2}]{k_f^{L2}} \text{RGL}$ | $k_f^{L2} = 2.78\ mM^{-1}\ ms^{-1}$<br>$k_b^{G1} = 2.78 \cdot 10^{-3}\ ms^{-1}$ | Alterred from (Falkenburger et al., 2010) |
| 4 | RL Binding to G | $\text{RL} + \text{G} \xrightleftharpoons[k_b^{G2}]{k_f^{G2}} \text{RLG}$ | $k_f^{G2} = 2.7 \cdot 10^{-5}\ \mu m^2\ ms^{-1}$<br>$k_b^{G2} = 6.8 \cdot 10^{-3}\ ms^{-1}$ | Alterred from (Falkenburger et al., 2010) |
| 5 | RL Binding to $G\beta\gamma$ | $\text{RL} + G\beta\gamma \xrightleftharpoons[k_b^{G2b}]{k_f^{G2b}} \text{RL}G\beta\gamma$ | $k_f^{G2b} = 2.7 \cdot 10^{-5}\ \mu m^2\ ms^{-1}$<br>$k_b^{G2b} = 6.8 \cdot 10^{-3}\ ms^{-1}$ | Alterred from (Falkenburger et al., 2010) |
| 6 | Nucleotide Exchange G | $\text{G} \xrightarrow{k^{NX\ RG}} G\beta\gamma + G\alpha{-}\text{GTP}$ | $k^{NX\ RG} = 1.5 \cdot 10^{-8}\ ms^{-1}$ | (Falkenburger et al., 2010) |
| 7 | Nucleotide Exchange RG | $\text{RG} \xrightarrow{k^{NX\ RG}} RG\beta\gamma + G\alpha{-}\text{GTP}$ | $k^{NX\ RG} = 1.5 \cdot 10^{-8}\ ms^{-1}$ | (Falkenburger et al., 2010) |
| 8 | Nucleotide Exchange RLG | $\text{RLG} \xrightarrow{k^{NX\ RLG}} RLG\beta\gamma + G\alpha{-}\text{GTP}$ | $k^{NX\ RLG} = 6.5 \cdot 10^{-4}\ ms^{-1}$ | (Falkenburger et al., 2010) |
| 9 | Nucleotide Exchange Phosphate | $G\alpha{-}\text{GDP} \xrightarrow{k^{GTPase1}} G\alpha{-}\text{GTP}$ | $k^{NX\ P} = 4.7 \cdot 10^{-3}\ ms^{-1}$ | (Falkenburger et al., 2010) |

| # | Reaction | Formula | Parameter Values | Source |
|---|---|---|---|---|
| 10 | GTPase1 | $G\alpha\text{–GTP} \xrightarrow{k^{GTPase1}} G\alpha\text{–GDP}$ | $k^{GTPase1} = 7.8 \cdot 10^{-5}\ ms^{-1}$ | Alterred from (Falkenburger et al., 2010) |
| 11 | GTPase2 | $G\alpha\text{–GTP–PLC} \xrightarrow{k^{GTPase2}} G\alpha\text{–GDP–PLC}$ | $k^{GTPase2} = 4.5 \cdot 10^{-2}\ ms^{-1}$ | Alterred from (Falkenburger et al., 2010) |
| 12 | PLC Association with $G\alpha$-GTP | $\text{PLC} + G\alpha\text{–GTP} \xrightarrow{k^{PLCassoc}} G\alpha\text{–GTP–PLC}$ | $k^{PLCassoc} = 5.0{\cdot}10^{-2}\ \mu m^2 ms^{-1}$ | Alterred from (Falkenburger et al., 2010) |
| 13 | PLC Dissociation from $G\alpha$-GDP | $G\alpha\text{–GDP–PLC} \xrightarrow{k^{PLCdiss}} G\alpha\text{–GDP} + \text{PLC}$ | $k^{PLCdiss} = 3.55 \cdot 10^{-3}\ ms^{-1}$ | Alterred from (Falkenburger et al., 2010) |
| 14 | G Protein Reconstitution | $G\beta\gamma + G\alpha\text{–GDP} \xrightarrow{k^{reconst}} \text{G}$ | $k^{reconst} = 1.0 \cdot 10^{-3}\ \mu m^2\ ms^{-1}$ | (Falkenburger et al., 2010) |
| 15 | Lipid 4-Kinase Activity | $\text{PI} \xrightarrow{k^{4K}} \text{PI(4) P}$ | $k^{4K} = 6.0 \cdot 10^{-5}\ ms^{-1}$ | Alterred from (Kruse et al., 2016) |
| 16 | Lipid 5-Kinase Activity | $\text{PI(4) P} \xrightarrow{k^{5K}} \text{PIP}_2$ | $k^{5K} = 5.0 \cdot 10^{-4}\ ms^{-1}$ | Alterred from (Kruse et al., 2016) |
| 17 | Lipid 4-Phosphotase Activity | $\text{PI(4) P} \xrightarrow{k^{4P}} \text{PI}$ | $k^{4P} = 3.0 \cdot 10^{-3}\ ms^{-1}$ | Alterred from (Kruse et al., 2016) |
| 18 | Lipid 5-Phosphotase Activity | $\text{PIP}_2 \xrightarrow{k^{5P}} \text{PI(4) P}$ | $k^{5P} = 7.0 \cdot 10^{-4}\ ms^{-1}$ | Alterred from (Kruse et al., 2016) |
| 19 | PLC Hydrolyis of $\text{PIP}_2$ | $\text{PIP}_2 \xrightarrow{k^{PLC}} \text{IP}_3 + \text{DAG}$ | $k^{PLC} = 0.03 * \text{G}\alpha\text{-GTP-PLC}$ | Alterred from (Kruse et al., 2016) |

| # | Reaction | Formula | Parameter Values | Source |
|---|---|---|---|---|
| 20 | $IP_5P$ Binding to $IP_3$ | $IP_5P + IP_3 \underset{k_b^{ip5p}}{\overset{k_f^{ip5p}}{\rightleftharpoons}} IP_5P-IP_3$ | $k_f^{ip5p} = 59.0\ mM^{-1}\ ms^{-1}$<br>$k_b^{ip5p} = 7.2 \cdot 10^{-2}\ ms^{-1}$ | (Doi et al., 2005) |
| 21 | $IP_2$ Formation | $IP_5P-IP_3 \xrightarrow{k^{ip2}} IP_5P + IP_2$ | $k^{ip2} = 1.8 \cdot 10^{-2}\ ms^{-1}$ | (Doi et al., 2005) |
| 22 | $IP_3$ Kinase Binding to Calcium | $IP_3K + 2\,Ca^{2+} \underset{k_b^{ip3k\ ca}}{\overset{k_f^{ip3k\ ca}}{\rightleftharpoons}} IP_3K-Ca_2$ | $k_f^{ip3k\ ca} = 1.11 \cdot 10^2\ mM^{-1} ms^{-1}$<br>$k_b^{ip3k\ ca} = 0.1\ ms^{-1}$ | (Doi et al., 2005) |
| 23 | $IP_3$ Kinase Binding to $IP_3$ | $IP_3K-Ca_2 + IP_3 \underset{k_b^{ip3k\ ip3}}{\overset{k_f^{ip3k\ ip3}}{\rightleftharpoons}} IP_3K-Ca_2-IP_3$ | $k_f^{ip3k\ ip3} = 5.0 \cdot 10^2\ mM^{-1}\ ms^{-1}$<br>$k_b^{ip3k\ ip3} = 8.0 \cdot 10^{-2}\ ms^{-1}$ | (Doi et al., 2005) |
| 24 | IP4 Formation | $IP_3K-Ca_2-IP_3 \xrightarrow{k^{ip4}} IP_3K-Ca_2 + IP_4$ | $k^{ip4} = 2.0 \cdot 10^{-2}\ ms^{-1}$ | (Doi et al., 2005) |
| 25 | Degradation of DAG | $DAG \xrightarrow{k^{DAGase}}$ | $k^{DAGase} = 2.0 \cdot 10^{-4}\ ms^{-1}$ | (Kruse et al., 2016) |
| 26 | $PIP_2$ Buffering | $PIP_2 \underset{k_b^{pip2}}{\overset{k_f^{pip2}}{\rightleftharpoons}} PIP_2bound$ | $k_f^{pip2} = 1.0 \cdot 10^{-3}\ \mu M^2\ ms^{-1}$<br>$k_b^{pip2} = 2.0 \cdot 10^{-3}\ ms^{-1}$ | (Falkenburger et al., 2013) |
| 27 | $PIP_2$ Binding to KCNQ Channels | $PIP_2 + KCNQ \underset{k_b^{kcnq}}{\overset{k_f^{kcnq}}{\rightleftharpoons}} PIP_2KCNQ$ | $k_f^{kcnq} = 5.0 \cdot 10^{-5}\ \mu M^2\ ms^{-1}$<br>$k_b^{kcnq} = 0.1\ ms^{-1}$ | (Falkenburger et al., 2013) |
| 28 | Calbindin-$D_{28k}$ Buffering | $CB + Ca^{2+} \underset{k_b^{cb}}{\overset{k_f^{cb}}{\rightleftharpoons}} CBCa$ | $k_f^{cb\ high} = 11.0\ mM^{-1}\ ms^{-1}$<br>$k_b^{cb\ high} = 2.607 \cdot 10^{-3}\ ms^{-1}$<br>$k_f^{cb\ low} = 8.7\ ms^{-1}$<br>$k_b^{cb\ low} = 3.57 \cdot 10^{-2}\ ms^{-1}$ | (Nägerl et al., 2000) |
| 29 | Calreticulin Buffering | $CALR + Ca^{2+} \underset{k_b^{calr}}{\overset{k_f^{calr}}{\rightleftharpoons}} CALRCa$ | $k_f^{calr} = 1.0 \cdot 10^{-2}\ mM^{-1}\ ms^{-1}$<br>$k_b^{calr} = 2.0 \cdot 10^{-2}\ ms^{-1}$ | (Doi et al., 2005) |
| 30 | Oregon Green BAPTA-1 binding | $OGB + Ca^{2+} \underset{k_b^{ogb1}}{\overset{k_f^{ogb1}}{\rightleftharpoons}} OGBCa$ | $k_f^{ogb1} = 10.0\ mM^{-1}\ ms^{-1}$<br>$k_b^{ogb1} = 4.3 \cdot 10^{-3}\ ms^{-1}$ | (Thomas et al., 2000) |

| # | Reaction | Formula | Parameter Values | Source |
|---|---|---|---|---|
| 31 | PMCA Calcium Binding | $\text{PMCA} + \text{Ca}^{2+} \underset{k_b^{pmca\ ca}}{\overset{k_f^{pmca\ ca}}{\rightleftharpoons}} \text{PMCACa}$ | $k_f^{pmca\ ca} = 2.5 \cdot 10^4\ mM^{-1}\ ms^{-1}$ $k_b^{pmca\ ca} = 2.0\ ms^{-1}$ | (Doi et al., 2005) |
| 32 | PMCA Extracellular Release of $\text{Ca}^{2+}$ | $\text{PMCACa} \xrightarrow{k^{pmca\ rel}} \text{PMCA}$ | $k^{pmca\ rel} = 5.0 \cdot 10^2\ ms^{-1}$ | (Doi et al., 2005) |
| 33 | $\text{IP}_3$ Receptor Opening | $\text{RI} + \text{Ca}^{2+} \underset{k_b^{ip3r\ 1}}{\overset{k_f^{ip3r\ 1}}{\rightleftharpoons}} \text{R}_{\text{Open}}$ | $k_f^{ip3r\ 1} = 8000.0\ mM^{-1}\ ms^{-1}$ $k_b^{ip3r\ 1} = 2.0 \cdot 10^{-3}\ ms^{-1}$ | (Doi et al., 2005) |
| 34 | $\text{IP}_3$ Receptor $\text{IP}_3$ Binding | $\text{R} + \text{IP}_3 \underset{k_b^{ip3r\ 2}}{\overset{k_f^{ip3r\ 2}}{\rightleftharpoons}} \text{RI}$ | $k_f^{ip3r\ 2} = 1000.0 \cdot 10^{-2}\ mM^{-1}\ ms^{-1}$ $k_b^{ip3r\ 2} = 25.8 \cdot 10^{-3}\ ms^{-1}$ | (Doi et al., 2005) |
| 35 | $\text{IP}_3$ Receptor Inactivation 1 | $\text{R} + \text{Ca}^{2+} \underset{k_b^{ip3r\ 3}}{\overset{k_f^{ip3r\ 3}}{\rightleftharpoons}} \text{RC}$ | $k_f^{ip3r\ 3} = 8.88 \cdot 10^{-3}\ mM^{-1} ms^{-1}$ $k_b^{ip3r\ 3} = 4.995 \cdot 10^{-3}\ ms^{-1}$ | (Doi et al., 2005) |
| 36 | $\text{IP}_3$ Receptor Inactivation 2 | $\text{RC} + \text{Ca}^{2+} \underset{k_b^{ip3r\ 4}}{\overset{k_f^{ip3r\ 4}}{\rightleftharpoons}} \text{RC}_2$ | $k_f^{ip3r\ 4} = 19.98\ mM^{-1}\ ms^{-1}$ $k_b^{ip3r\ 4} = 9.99 \cdot 10^{-2}\ ms^{-1}$ | (Doi et al., 2005) |
| 37 | $\text{IP}_3$ Receptor Inactivation 3 | $\text{RC}_2 + \text{Ca}^{2+} \underset{k_b^{ip3r\ 5}}{\overset{k_f^{ip3r\ 5}}{\rightleftharpoons}} \text{RC}_3$ | $k_f^{ip3r\ 5} = 39.95\ mM^{-1}\ ms^{-1}$ $k_b^{ip3r\ 5} = 14.985 \cdot 10^{-2}\ ms^{-1}$ | (Bicknell and Goodhill, 2016) |
| 38 | $\text{IP}_3$ Receptor Inactivation 4 | $\text{RC}_3 + \text{Ca}^{2+} \underset{k_b^{ip3r\ 5}}{\overset{k_f^{ip3r\ 5}}{\rightleftharpoons}} \text{RC}_4$ | $k_f^{ip3r\ 5} = 59.94\ mM^{-1}\ ms^{-1}$ $k_b^{ip3r\ 5} = 19.98 \cdot 10^{-2}\ ms^{-1}$ | (Doi et al., 2005) |
| 39 | Calcium flux into ER due to SERCA | $Ca_{cyt}^{2+} \xrightarrow{k_f^S} Ca_{ER}^{2+}$ | $k_f^S = \frac{g_S \cdot [Ca_{cyt}^{2+}]^2}{[Ca_{cyt}^{2+}]^2 + 0.0013^2}$ $g_S = 6.35 \cdot 10^4\ mM^{-1}\ ms^{-1}$ | Alterred from (Falkenburger et al., 2013) |
| 40 | Leak from ER into Cytosol | $Ca_{ER}^{2+} \xrightarrow[k_b^{leak\ ER}]{k_f^{leak\ ER}} Ca_{cyt}^{2+}$ | $k_f^{leak\ ER} = 2.1 \cdot 10^3\ mM^{-1} ms^{-1}$ | Tuned Value |

| # | Reaction | Formula | Parameter Values | Source |
|---|---|---|---|---|
| 41 | Leak from Extracellular into Cytosol | $dCa^{2+}_{cyt} = g_{leak\ ext} \cdot (Ca_{ext} - Ca_{cyt})$ | $g_{leak\ ext} = 3.2 \cdot 10^{-5}$ | Tuned Value |
| 42 | Simulated Fluorescence | $F = f_{mult} \cdot OGB1Ca + OGB1$ | $f_{mult} = 14.0$ | (Molecular Probes, 2005) |
| 43 | Store Operated Calcium Entry | $dCa^{2+}_{ER} = g_{SOCE} \cdot \ln(1 + e^{v_m - v_{init}}) \cdot e^{\frac{-(Ca_{ER} - Ca_d)}{k_{SOCE}}}$ | $g_{SOCE} = 5.0 \cdot 10^{-4}$<br>$v_{init} = -65.0mV$<br>$Ca_d = 0.1mM$<br>$k_{SOCE} = 0.015$ | Tuned Values |

Table S2: Table of mechanism kinetic parameters, conductances, and diffusion, with their respective sources

## REFERENCES


Bicknell, B. A. and Goodhill, G. J. (2016). Correction for Bicknell and Goodhill, Emergence of ion channel modal gating from independent subunit kinetics. *Proceedings of the National Academy of Sciences* 113, E8357–E8357. doi:10.1073/pnas.1619297114

Doi, T., Kuroda, S., Michikawa, T., and Kawato, M. (2005). Inositol 1,4,5-Trisphosphate-Dependent Ca2+ Threshold Dynamics Detect Spike Timing in Cerebellar Purkinje Cells. *Journal of Neuroscience* 25, 950–961. doi:10.1523/JNEUROSCI.2727-04.2005

Falkenburger, B. H., Dickson, E. J., and Hille, B. (2013). Quantitative properties and receptor reserve of the DAG and PKC branch of Gq-coupled receptor signaling. *J Gen Physiol* 141, 537–555. doi:10.1085/jgp.201210887

Falkenburger, B. H., Jensen, J. B., and Hille, B. (2010). Kinetics of M1 muscarinic receptor and G protein signaling to phospholipase C in living cells. *The Journal of General Physiology* 135, 81–97. doi:10.1085/jgp.200910344

Kruse, M., Vivas, O., Traynor-Kaplan, A., and Hille, B. (2016). Dynamics of Phosphoinositide-Dependent Signaling in Sympathetic Neurons. *Journal of Neuroscience* 36, 1386–1400. doi:10.1523/JNEUROSCI.3535-15.2016

[Dataset] Molecular Probes (2005). Long-Wavelength Calcium Indicators. `https://assets.thermofisher.com/TFS-Assets/LSG/manuals/mp03010.pdf`

Müller, A., Kukley, M., Stausberg, P., Beck, H., Müller, W., and Dietrich, D. (2005). Endogenous Ca2+ Buffer Concentration and Ca2+ Microdomains in Hippocampal Neurons. *Journal of Neuroscience* 25, 558–565. doi:10.1523/jneurosci.3799-04.2005

Nägerl, U. V., Novo, D., Mody, I., and Vergara, J. L. (2000). Binding Kinetics of Calbindin-D28k Determined by Flash Photolysis of Caged Ca2+. *Biophysical Journal* 79, 3009–3018. doi:10.1016/S0006-3495(00)76537-4

Oh, M. M., Oliveira, F. A., Waters, J., and Disterhoft, J. F. (2013). Altered calcium metabolism in aging CA1 hippocampal pyramidal neurons. *J Neurosci* 33, 7905–7911. doi:10.1523/JNEUROSCI.5457-12.2013

Power, J. M. and Sah, P. (2002). Nuclear Calcium Signaling Evoked by Cholinergic Stimulation in Hippocampal CA1 Pyramidal Neurons. *The Journal of Neuroscience* 22, 3454–3462. doi:20026335

Solovyova, N., Veselovsky, N., Toescu, E. C., and Verkhratsky, A. (2002). Ca2+ dynamics in the lumen of the endoplasmic reticulum in sensory neurons: direct visualization of Ca2+-induced Ca2+ release triggered by physiological Ca2+ entry. *European Molecular Biology Organization Journal* 21, 622–630. doi:10.1093/emboj/21.4.622

Thomas, D., Tovey, S. C., Collins, T. J., Bootman, M. D., Berridge, M. J., and Lipp, P. (2000). A comparison of fluorescent Ca2+ indicator properties and their use in measuring elementary and global Ca2+ signals. *Cell Calcium* 28, 213–223. doi:10.1054/ceca.2000.0152
